# Cancer cell migration on elongate protrusions of fibroblasts in collagen matrix

**DOI:** 10.1038/s41598-018-36646-z

**Published:** 2019-01-22

**Authors:** Kaoru Miyazaki, Jun Oyanagi, Daisuke Hoshino, Shinsaku Togo, Hiromichi Kumagai, Yohei Miyagi

**Affiliations:** 1 Molecular Pathology and Genetics Division, Kanagawa Cancer Center Research Institute, 2-3-2 Nakao, Asahi-ku, Yokohama, 241-8515 Japan; 2 Division of Cell Biology, Kihara Institute for Biological Research, Yokohama City University, 641-12 Maioka-cho, Totsuka-ku, Yokohama, 244-0813 Japan; 3 Cancer Cell Biology Division, Kanagawa Cancer Center Research Institute, 2-3-2 Nakao, Asahi-ku, Yokohama, 241-8515 Japan; 4 Division of Respiratory Medicine, Juntendo University of Medicine, 3-1-3 Hongo, Bunkyo-Ku, Tokyo, 113-8431 Japan; 5 Kumagai Fellow laboratory, Innovative Technology Research Center, Technology General Division, AGC Inc, 1150 Hazawa-cho, Kanagawa-ku, Yokohama, 221-8515 Japan; 6 Present Address: Internal Medicine III, Wakayama Medical University, 811-1 Kimiidera, Wakayama, 641-8509 Japan

## Abstract

Cancer-associated fibroblasts (CAFs) play critical roles in the tumor progression. However, it remains unclear how cancer cells migrate in the three-dimensional (3D) matrix of cancer tissues and how CAFs support the cancer invasion. Here we propose a novel mechanism of fibroblast-dependent cancer cell invasion in the 3D collagen matrix. Human cancer cell lines from the pancreas (Panc-1), lung (A549) and some other organs actively adhered to normal fibroblasts and primary lung CAFs in cultures. To show its significance in tumor invasion, we designed a new invasion assay in which homogeneous microspheroids consisting of cancer cells and fibroblasts were embedded into collagen gel. Time-lapse experiments showed that cancer cells adhered to and quickly migrated on the long protrusions of fibroblasts in the 3D collagen matrix. Fibroblast-free cancer cells poorly invaded the matrix. Experiments with function-blocking antibodies, siRNAs, and immunocytochemistry demonstrated that cancer cells adhered to fibroblasts through integrin α5β1-mediated binding to fibronectin on the surface of fibroblasts. Immunochemical analyses of the co-cultures and lung cancers suggested that cancer cells could acquire the migratory force by the fibronectin/integrin signaling. Our results also revealed that the fibroblast-bound fibronectin was a preferential substrate for cancer cells to migrate in the collagen matrix.

## Introduction

During malignant progression of cancer, basement membranes surrounding cancer cells disappear due to the proteolytic degradation and impaired synthesis of the matrix proteins. This event allows cancer cells to directly interact with a variety of stromal components. It has well been established that complicate interaction between cancer cells and their microenvironment plays critical roles in the tumor progression such as aggressive growth, invasion and metastasis^1,2^. The tumor microenvironment is constituted of many types of stromal cells including fibroblasts, vascular endothelial cells and inflammatory cells, extracellular matrices (ECMs), and many kinds of soluble factors. Fibroblasts are the most abundant and most critical cell type for cancer progression^3–5^. Myofibroblasts and other populations of activated fibroblasts in the tumor microenvironment are called cancer-associated fibroblasts (CAFs). They stimulate tumor cell progression and invasion *in vitro*^6^ and tumor growth and angiogenesis *in vivo*^7,8^. The mutual interaction between cancer cells and CAFs supports the cancer progression in many ways. Growth factors, cytokines, ECMs and the matrix-degrading enzymes MMPs, all of which are induced by such interaction, are obviously required for the tumor growth, invasion and metastasis^3,4,9^. For example, fibroblast-derived HGF facilitates the growth and invasive activity of cancer cells^10^, while TGF-β induces the epithelial to mesenchymal transition (EMT) of cancer cells, a key step to the invasive cancer cells^11^ and converts normal fibroblasts to the activated fibroblasts, *i.e*. myofibroblasts or CAFs^12^. ECM components such as collagens^13,14^, laminins^15^ and fibronectin^16,17^ are important substrates for cancer cells to grow, survive and migrate in tissues. Fibronectin is a major ECM component produced by fibroblasts, and its ED-A isoform is upregulated in cancer stroma^17^. Recent studies have shown that CAF-derived fibronectin matrix guides cancer cell migration^18–20^.

Although many past studies extensively revealed the roles of various microenvironmental factors in tumor progression, it has not been fully understood yet how cancer cells migrate through the three-dimensional (3D) matrix and how CAFs support the cancer invasion. To address these issues, studies in 3D conditions are critically important. A recent study demonstrated that fibroblasts lead collective cell migration of epidermoid carcinoma cells in collagen matrix by the direct cell adhesion through E-cadherin/N-cadherin heterophilic interaction^21^. Relatively less attention has been paid on the significance of the direct interaction between cancer cells and CAFs in tumor progression^22–25^. As cancer cells often lose E-cadherin on the cell surface during their malignant progression, the E-/N-cadherin mechanism seems irrelevant to many kinds of invasive cancers^26–28^. Our recent study showed that fibroblasts induce EMT-like morphological change of cancer cells on collagen gels^29^. Further attempt to clarify this mechanism has revealed that the heterotypic cell adhesion between cancer cells and fibroblasts facilitates the morphological EMT of the cancer cells and their migration. The present study demonstrates a tumor invasion mechanism in which cancer cells invade the collagen matrix by binding to fibroblasts through the integrin/fibronectin interaction.

## Results

### Direct interaction of various types of human cancer cells with normal fibroblasts or CAFs in two-dimensional (2D) cultures

To investigate the direct interaction between human cancer cells and fibroblasts, we mainly used co-culture models of GFP-labeled Panc-1 pancreatic adenocarcinoma cell line or GFP-labeled A549 lung adenocarcinoma cell line with the fetal lung fibroblast line WI-38 or the OUS-11 fibroblast line derived from a non-neoplastic tissue of a lung adenocarcinoma. We also used primary lung CAFs (LuCAFs) and control normal fibroblasts (Ctr-NLFs) from the same adenocarcinoma patient. The expression of the myofibroblast marker αSMA was much higher in WI-38 and OUS-11 than Ctr-NLF and LuCAF as analyzed by immunoblotting, but it was comparable among the four cell lines in immunocytochemistry, indicating that the cytoskeletal αSMA level does not necessarily reflect its total protein level (Fig. S1a,b). When compared between Ctr-NLF and LuCAF, the latter appeared slightly higher than the former in both analyses. All fibroblast lines expressed fibronectin at comparable levels (Fig. S1a,c). These results suggested that all these cell line had been activated in culture.

**Figure 1:**
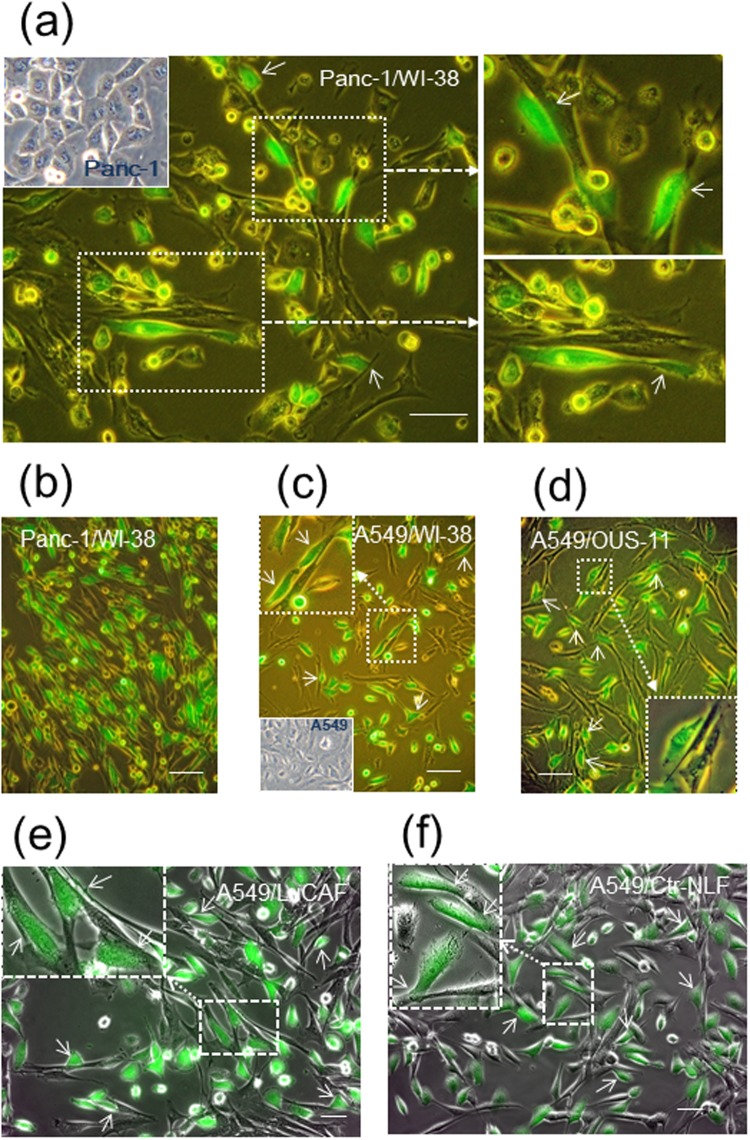
Adhesion of Panc-1 and A549 adenocarcinoma cells (green) to different kinds of fibroblasts in 2D cultures. GFP-labeled cancer cells (green) were co-cultured with the following fibroblasts in 2D conditions for the indicated lengths of time, and their phase-contrast images with GFP signals were obtained. (**a**) Panc-1 and WI-38 cells incubated overnight. (**b**) Panc-1/WI-38 (4 days). (**c**) A549/WI-38 (2 days). (**d**) Panc-1/OUS-11 (3 days). (**e**) A549/LuCAF (2 days). (**f**) A549/Ctr-NLFs (2 days). Broken arrows indicate magnified views of the boxed fields, and small arrows point cancer cells stretched or elongated by binding to fibroblasts. Insets in (**a**,**c**) indicate morphology of Panc-1 and A549 cells in single cultures, respectively. Scale bars, 100 μm in (**a**–**d**) and 50 μm in (**e**,**f**).

When Panc-1 cells and WI-38 cells were mixed and plated on plastic dishes in 2D conditions, cancer cells actively bound to fibroblasts by extending pseudopodial protrusions during incubation for 20 h (Fig. 1a). Cancer cells contacted not only the lateral side of fibroblasts but also their apical surface (Fig. 1a, upper right panel). On further incubation, most of Panc-1 cells, like EMT-induced cells, exhibited extremely elongated shapes along the fibroblast structures (Fig. 1b). When Panc-1 cells were plated on a sub-confluent or confluent culture of WI-38 cells which had been pre-cultured for 2 or 3 days, such morphological changes of Panc-1 cells occurred in a few hours (Fig. S2ab and Video 1). The time-lapse video showed that cancer cells migrated by extending lamellipodia-like protrusions while keeping contact with fibroblasts. A549 lung adenocarcinoma cells also showed the heterotypic cell-cell interaction with WI-38 cells (Fig. 1c) and the normal lung fibroblast line OUS-11 (Fig. 1d). As shown by the time-lapse video, A549 cells more rapidly migrated along the fibroblast structures than Panc-1 cells (Video 2, see also Video 1). In an orthotopic lung cancer model, A549 cells interacted with the lung CAFs (LuCAFs) and the control fibroblasts (Ctr-NLFs) (Fig. 1e,f). There was no significant difference in the interaction with A549 cells between LuCAFs and Ctr-NLFs.

The heterotypic cell adhesion with WI-38 fibroblasts was also found in many types of invasive human cancer cells such as MKN-45 gastric carcinoma cells (Fig. S2c), STKM-1 gastric carcinoma cells (Fig. S2d), MIA-PaCa-2 pancreatic carcinoma cells (Fig. S2e) and MDA-MB-231 breast carcinoma cells (Fig. S2f). Although MKN-45 and STKM-1 cells hardly spread on the usual plastic surface, they could spread in contact with fibroblasts (Fig. S2c,d). Especially, STKM-1 cells attached to fibroblasts by extending extremely long protrusions. On the other hand, another group of cancer cell lines, such as MCF-7 breast adenocarcinoma (Fig. S3a,b), A431 vulval epidermoid carcinoma (Fig. S3c,d), CaSki squamous cell carcinoma (Fig. S3e) and MKN-74 gastric carcinoma (Fig. S3f), all of which expressed E-cadherin at high levels, formed tumor cell islands surrounded by fibroblasts in the co-cultures, suggesting stronger homophilic intercellular junctions compared with the heterophilic ones.

### Invasion of cancer cells in the presence of fibroblasts in three-dimensional (3D) collagen matrix

**Figure 2:**
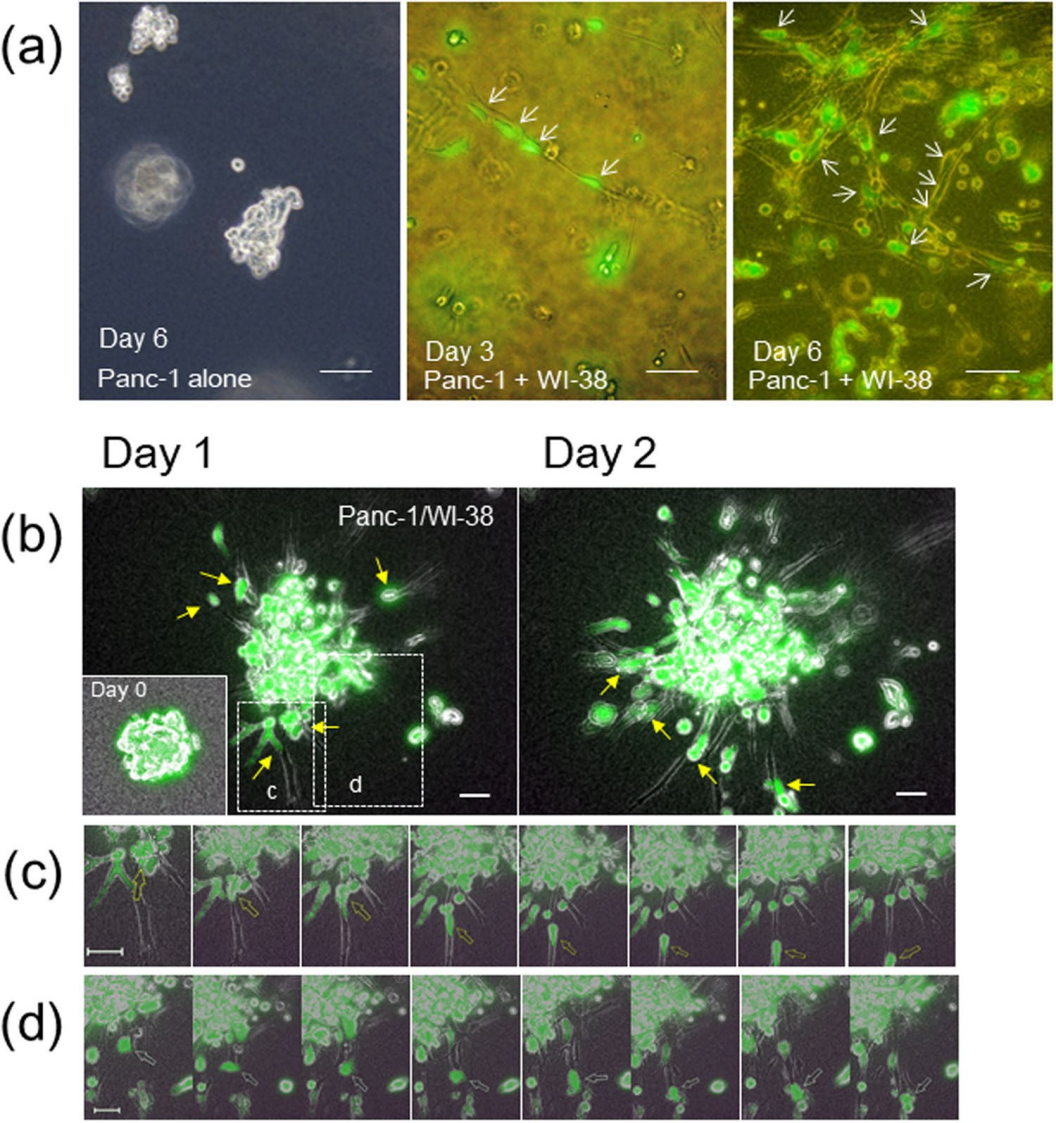
Panc-1 cells actively adhere to and migrate on strings of fibroblast protrusions in 3D collagen matrix. (**a**) Panc-1 cells (green) without (left) or with (center and right) WI-38 cells were inoculated and incubated in collagen gel. Scale bars, 100 μm. (**b**) Chimeric spheroids of Panc-1 and WI-38 cells were incubated in collagen gel. Fluorescent mages after 20 h (day 1) (left panel) and 40 h (day 2) (right panel) in incubation are shown. Inset at Day 1, the original spheroid at day 0. Scale bars, 50 μm. (**c**,**d**) Magnified time-lapse images in 30-min intervals of the boxed fields in (**b)** after 20-h (**c**) or 33-h (**d**) incubation. Arrows trace one cancer cell migrating on a fibroblast (**c**) or leaving a fibroblast (**d**). See also Video 3. In (**a**) and (**b**), small arrows point cancer cells binding to fibroblasts.

The 3D-collagen matrix culture was used to investigate a possible role of the cancer cell/fibroblast interaction in tumor invasion. When Panc-1 cells were embedded into the collagen gel, they kept round morphology or cell aggregates for over 6 days (Fig. 2a, left panel). However, when co-cultured with fibroblasts for a few days, cancer cells adhered to a string structure of fibroblasts (Fig. 2a, center panel). On further incubation, fibroblasts formed a net-work structure of their strings to which many cancer cells attached and extended protrusions (Fig. 2a, right panel). These observations suggested that the direct binding of cancer cells to fibroblasts would support cancer cell invasion in the 3D collagen matrix. It is also noted that fibroblasts extremely elongated, extending invasive protrusions in the 3D matrix.

To facilitate cancer cell invasion in the collagen matrix, we designed a new invasion assay in which homogeneous chimeric spheroids consisting of cancer cells and normal fibroblasts or CAFs were prepared in micro-fabricated 96-well plates and embedded into collagen gel. Panc-1 cells alone formed loose aggregates in the plates (Fig. S4a), but they produced solid chimeric spheroids with WI-38 cells in which cancer cells distributed in both the surface area and inner core of spheroids (Fig. S4b–d).

**Figure 3:**
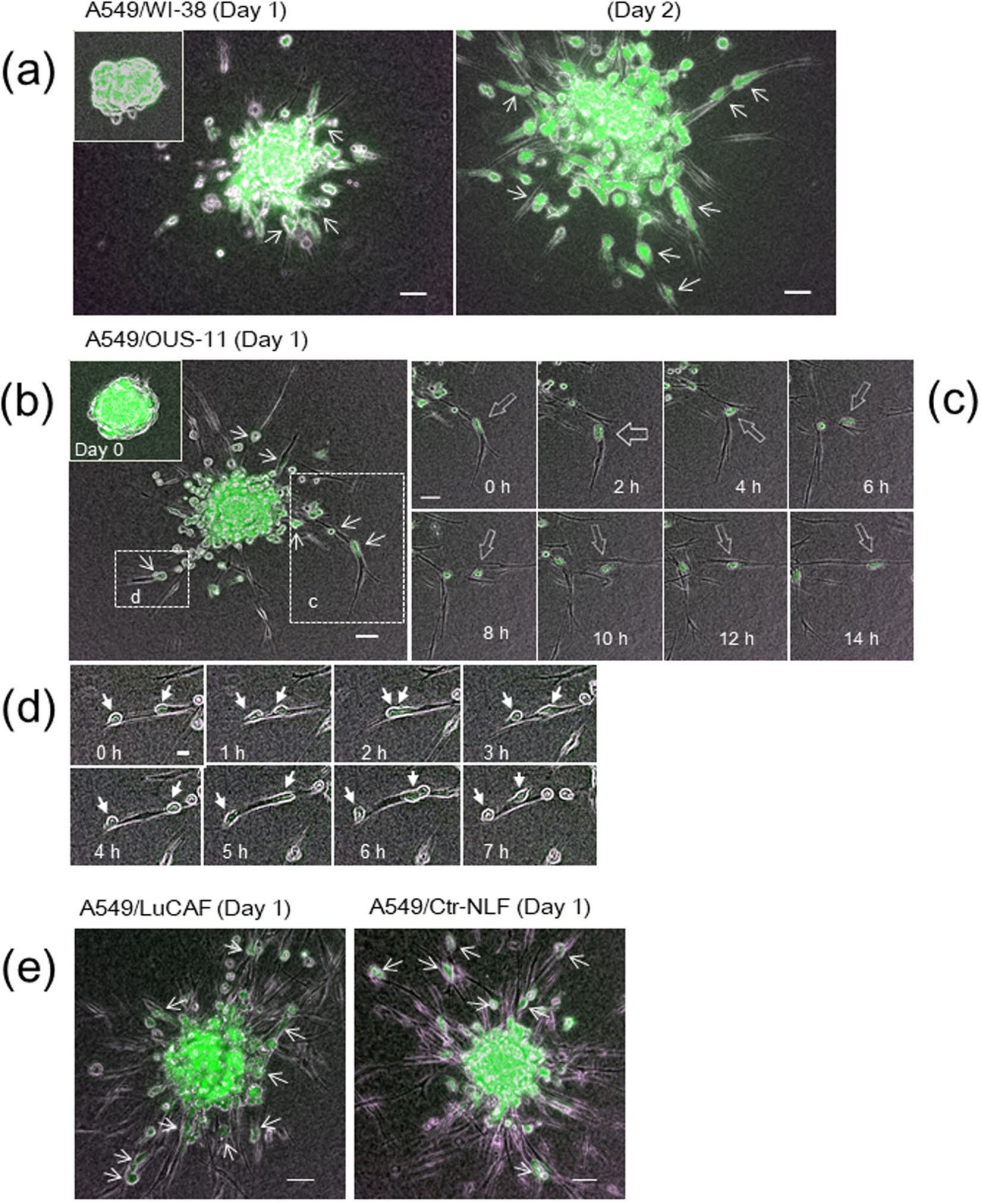
Collagen gel invasion of A549 cells (green) from spheroids with different types of fibroblasts. (**a**) A549/WI-38 spheroid incubated for 22 h (day 1) (left panel) and 44 h (day 2) (right panel). Inset, the original spheroid at day 0. (**b**) A549/OUS-11 spheroid incubated for 22 h (day 1). Inset, day 0. (**c**) Time-lapse images of the box “c” in (**b**) Arrows trace one cancer cell migrating on four fibroblasts in sequence. See Video 4. (**d**) Time-lapse images of the box “d” in (**b**) after 25-h incubation. Arrows trace two cancer cells showing reciprocating movement on a string of fibroblast. (**e**) A549/LuCAF (left panel) and A549/Ctr-NLF (right panel) spheroids after 22-h incubation (day 1). Note that both LuCAF and Ctr-NLF migrate very actively in the collagen matrix compared with WI-38 (**a**) and OUS-11 (**b**) at Day 1. Scale bars, 50 μm, except for 20 μm in (**d**) In all images, small arrows point cancer cells binding to fibroblasts.

When the Panc-1/WI-38 chimeric spheroids were embedded in the 3D collagen matrix, the cancer cell invasion began in a few hours and became evident in one or two days (Fig. 2b). After fibroblasts sufficiently invaded the collagen matrix in the radial direction from the spheroids, cancer cells attached to and migrated on the fibroblast protrusions, like trains running on a railway line (Fig. 2c and Video 3). The cancer cells on the fibroblast strings sometimes reversed the direction, while some cells jumped out of the fibroblasts into the collagen matrix at the tip of strings (Fig. 2d and Video 3). Aggregates of Panc-1 cells in the absence of fibroblasts rarely showed invasive morphology compared with those in their presence (Fig. S4e). When the chimeric spheroids of A549 cells and WI-38 cells were embedded in the collagen gel, the lung cancer cells showed fibroblast-dependent invasion more actively than Panc-1 cells (Fig. 3a). More striking invasion of both cancer cells and fibroblasts was observed in a combination of A549 and OUS-11 (Fig. 3b and Video 4). Cancer cells actively migrated while transferring fibroblasts one by one (Fig. 3c and Video 4). Some cancer cells that were unable to leave the fibroblast vehicle showed reciprocating movement on a string of fibroblast (Fig. 3d and Video 4). A strong invasive activity of A549 cells was also found when their chimeric spheroids with the primary lung CAFs (LuCAFs) or the control fibroblasts (Ctr-NLFs) were applied to the 3D invasion assay as the orthotopic lung cancer model (Fig. 3e and Video 5). Although cancer cells appeared to attach to LuCAFs more than Ctr-NLFs, the difference was not evident. When the four fibroblast lines were compared in the combination with A549 cells, LuCAFs and Ctr-NLFs appeared to have the highest invasive activity in the 3D invasion assay. In the case of OUS-11 fibroblasts, the invasion of fibroblasts was greater in the presence of A549 cells than Panc-1 cells, suggesting that cancer cells also affect invasive activity of fibroblasts (Fig. S4f).

All these observations strongly suggest that cancer cells acquire invasive activity by directly binding to fibroblasts in the 3D collagen matrix.

### Identification of molecules responsible for heterotypic intercellular adhesion

**Figure 4:**
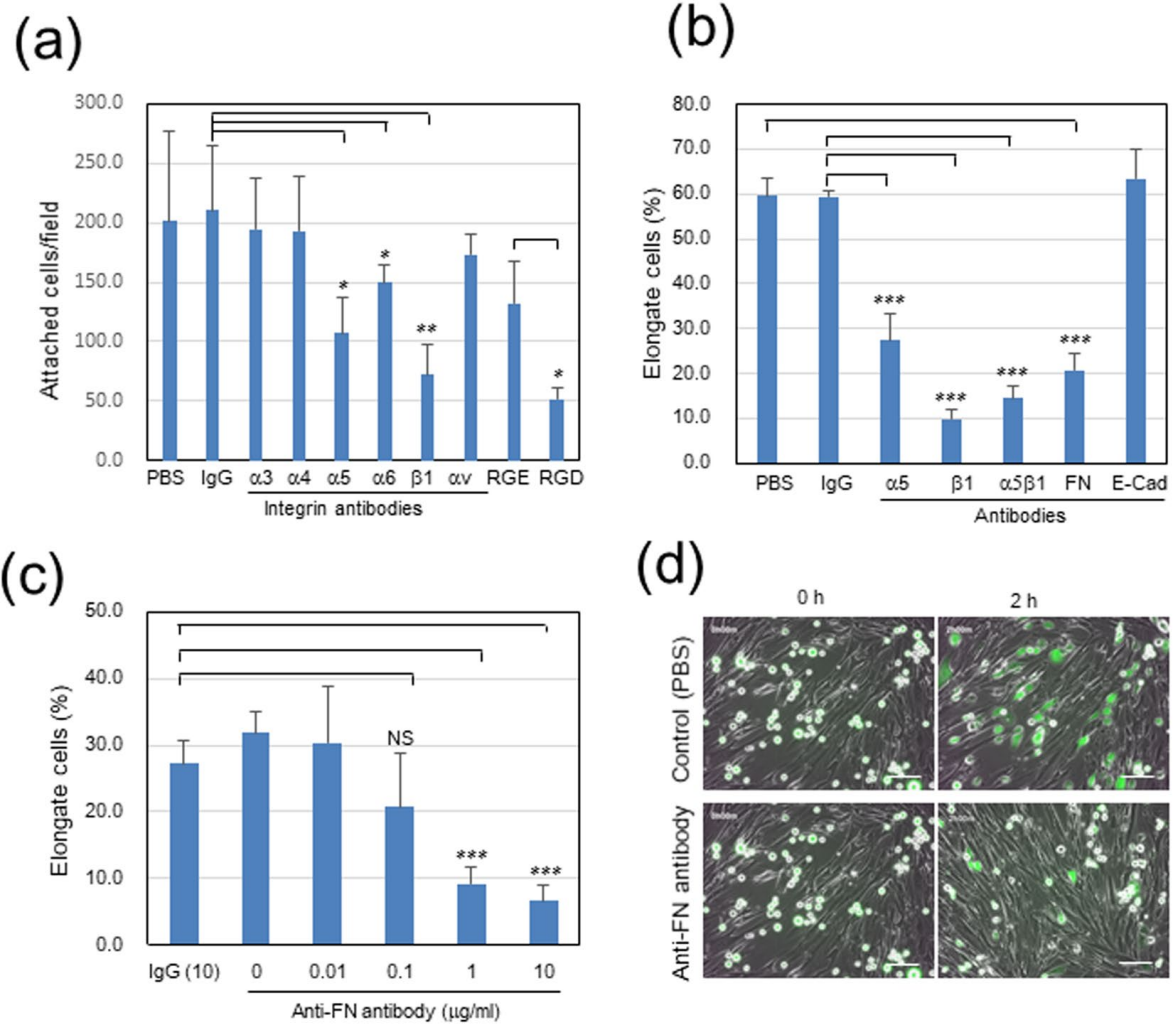
Inhibitory activities of anti-integrin antibodies and oligopeptides on adhesion of Panc-1 cells to WI-38 fibroblasts in 2D co-cultures. The anti-fibronectin (FN) antibody FN12-8 was applied to WI-38 cells, while the others were to Panc-1 cells. RGD and RGE peptides were used at 0.3 mM. (**a**) Panc-1 cell attachment to confluent WI-38 cells. Each column indicates the mean of the number of attached cells per field ± SD in triplicate wells. See also Fig. S5a. (**b**,**c**) Panc-1 cell elongation in contact with WI-38 cells. Panc-1 cells were incubated on sub-confluent cultures of WI-38 cells for 2 h, and total and elongate Panc-1 cells in contact with WI-38 cells were counted on the fluorescence images. Each column indicates the mean of the percentage of elongate cells ± SD in triplicate wells. ***p < 0.001. See also Fig. S5b,c. (**c**) Dose-dependent effect of anti-FN antibody (μg/ml). NS, not significant. (**d**) Representative images at 10 μg/ml FN antibody. Scale bars, 100 μm. See Videos 1 and 6. Other experimental conditions are described in Materials and Methods.

The molecular mechanism by which cancer cells directly binds to fibroblasts was investigated using the Panc-1/WI-38 co-culture model. Past studies showed the involvement of some integrins in the heterotypic adhesion of gastric cancer cells to CAFs^23^ or CAF-guided cancer cell migration^22^. Therefore, we first examined effects of function-blocking antibodies against some integrin molecules and the RGD peptide (integrin α5 inhibitor) on the Panc-1 cell attachment to the confluent culture of WI-38 cells. When Panc-1 cells were pretreated with each of these inhibitors, their attachment was more significantly inhibited by RGD and antibodies against integrin β1 and integrin α5 than the others (Figs 4a and S5a). This suggested that integrin α5β1 in cancer cells might bind to fibronectin on the surface of WI-38 cells. To verify this possibility, function-blocking antibodies were further tested using sub-confluent WI-38 cell cultures. In the assay, we determined the proportion of elongated or extended Panc-1 cells to the total cells in contact with WI-38 cells. The antibodies against integrin α5, β1 and α5β1 strongly inhibited the adhesion of cancer cells to fibroblasts (Figs 4b and S5b). Similarly, when WI-38 cells were pretreated with a function-blocking antibody against fibronectin, the heterotypic adhesion of Panc-1 cells to WI-38 cells was dose-dependently blocked (Fig. 4b–d and Video 6). Although E-/N-cadherin interaction has been reported in the adhesion of A431 epidermoid carcinoma cells to CAFs^21^, a function-blocking antibody against E-cadherin, which completely blocked the homotypic cell-cell adhesion of MCF-7 cells (Fig. S5c), did not inhibit the adhesion of Panc-1 cells to fibroblasts at all (Figs 4b and S5b).

**Figure 5:**
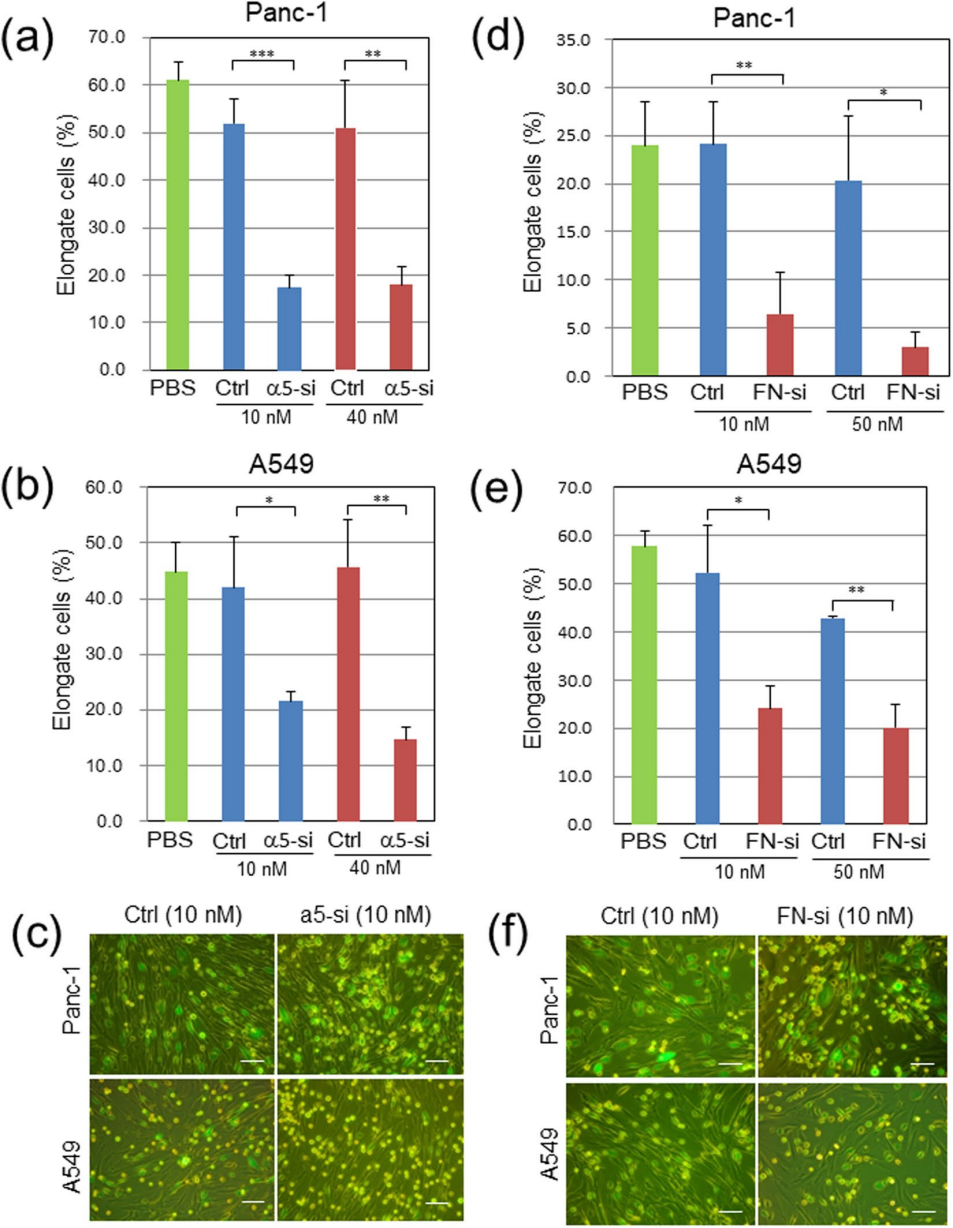
Depletion of integrin α5 in Panc-1 or A549 cells or fibronectin (FN) in WI-38 fibroblasts suppresses their heterotypic adhesion in 2D co-cultures. GFP-labeled cancer cells were transfected with control RNA (Ctrl) or a pool of α5-specific RNAs (α5-si), while WI-38 cells with control (Ctrl) or FN-specific RNAs (FN-si). Three-days later, cell adhesion assays with non-treated counterparts were done as described in Fig. 4b. (**a**,**c**) Ctrl- or α5-si-treated Panc-1 cells. (**b**,**c**) Ctrl- or α5-si-treated A549 cells. (**d**,**f**) Panc-1 cells and Ctrl- or FN-si-treated WI-38 cells. (**e**,**f**) A549 cells and Ctrl- or FN-si-treated WI-38 fibroblasts. (**c**,**f**) Representative images from the triplicate wells. *p < 0.05, **p < 0.01 and ***p < 0.001. Scale bars, 100 μm.

In addition to the experiments with the function-blocking antibodies, we performed knockdown experiments of integrin α5 and fibronectin genes with specific siRNAs. The treatment of Panc-1 and A549 cells with the specific siRNA pool efficiently blocked the expression of integrin α5 protein in both types of cancer cells as analyzed by immunoblotting (Fig. S6a). The knockdown of integrin α5 gene in Panc-1 and A549 cells significantly inhibited their elongation in contact with WI-38 fibroblasts (Fig. 5a–c). Similarly, the siRNA pool for fibronectin strongly blocked the fibronectin expression in WI-38 cells (Fig. S6b), and this treatment efficiently blocked the spreading of Panc-1 or A549 cancer cells in contact with WI-38 fibroblasts (Fig. 5d–f). The efficient suppression of fibronectin expression in WI-38 cells was confirmed by immunofluorescent staining for cell-associated fibronectin (Fig. S7a). The fibronectin knockdown also caused significant enlargement of WI-38 cells (Fig. S7b). On the other hand, the knockdown of integrin α5 had no significant effect on the morphology of Panc-1 cells (Fig. S7c).

**Figure 6:**
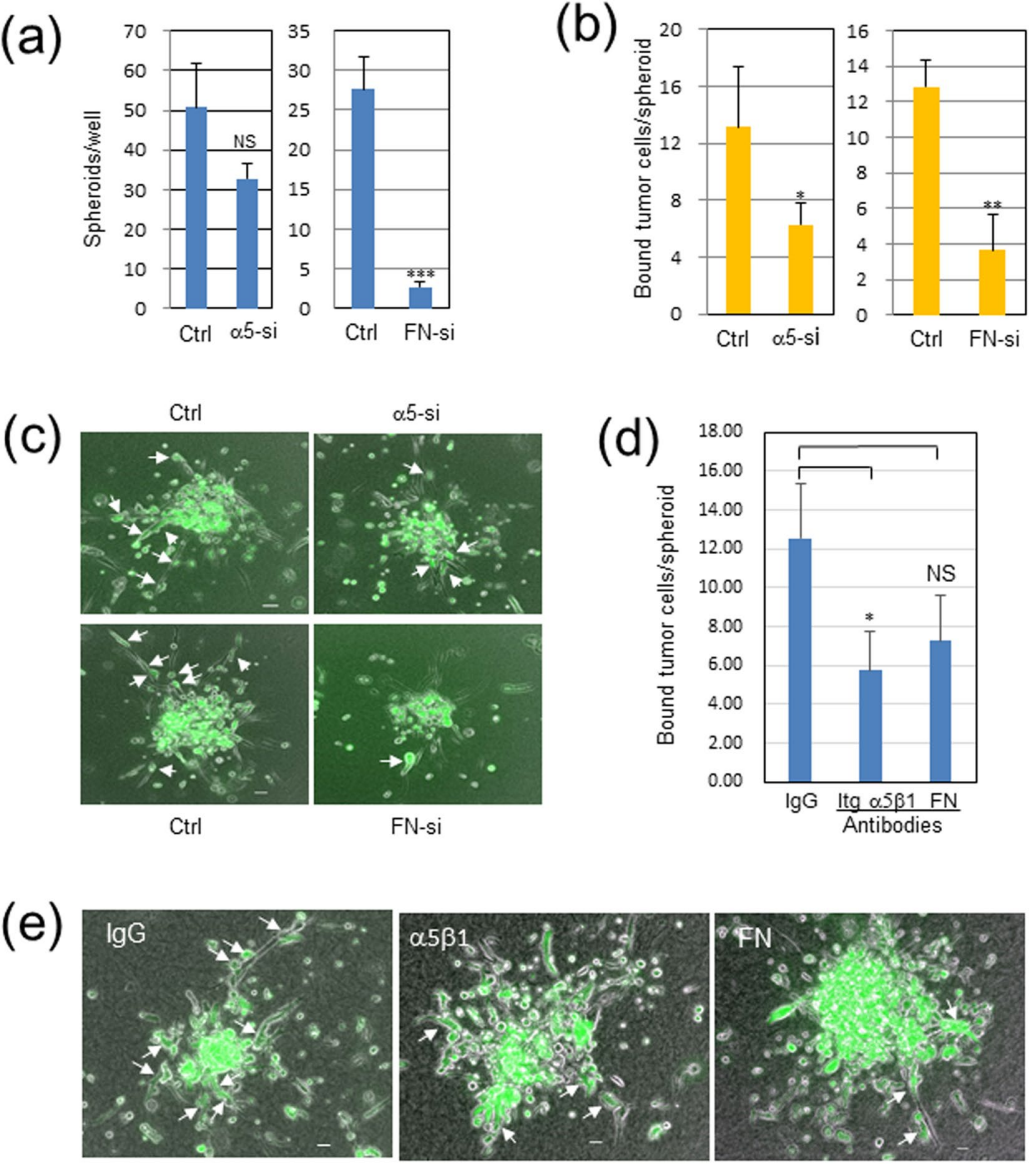
Knockdown or antibody treatment of integrin (Itg) α5 in Panc-1 cells or fibronectin (FN) in WI-38 fibroblasts reduces fibroblast-dependent Panc-1 cell invasion in 3D collagen gel. Panc-1 cells and WI-38 cells were transfected with 10 nM control or specific siRNAs for Itg α5 or FN, respectively. These cells were harvested on the next day, mixed with non-treated counterparts, and incubated on an EZSPERE plate for 20 h. Chimeric spheroids thus produced were used in the following analyses. (**a**) Chimeric spheroid formation in α5-si Panc-1 (left panel) or FN-si WI-38 (right panel). Ordinate, the mean of the spheroid number per well ± SD in triplicate wells. NS, not significant (*p* = 0.056); ***p < 0.001. (**b**,**c**) Panc-1 cell invasion in contact with WI-38 cells in α5-si Panc-1 (left panel) or FN-si WI-38 (right panel). The above chimeric spheroids were incubated in collagen gel for 2 days, and Panc-1 cells elongated in contact with invading WI-38 cells, shown by arrows in (**c**), were counted for each spheroid. Ordinate, the mean of the number of elongate Panc-1 cells per spheroid ± SD in triplicate wells. The total spheroids analyzed were 14 in Ctr and α5-si Panc-1, 12 in Ctr WI-38, and 7 in FN-si WI-38. *p < 0.05, **p < 0.01. (**c**) Representative mages. Scale bars, 100 μm. See also Video 7. (**d**) Suppressive effects of anti-Itg-α5β1 and anti-FN antibodies on Panc-1 cell invasion. Invading Panc-1 cells in contact with fibroblasts were measured as above for each spheroid (total 6 to 8 spheroids per group). *p* = 0.066 in FN-si. (**e**) Representative images in (**d**).

The knockdown effects of the integrin α5 and fibronectin genes were evaluated for the Panc-1/WI-38 interaction in the EZSPHERE micro-fabricated vessels. The integrin knockdown in Panc-1 cells tended to reduce the number of the chimeric spheroids produced with WI-38 cells (*p* = 0.056) (Fig. 6a, left panel), while the fibronectin knockdown in WI-38 cells greatly inhibited the spheroid formation (*p* < 0.001) (Fig. 6a, right panel). In addition, the sizes of the chimeric spheroids were weakly but significantly reduced by both the knockdown of integrin α5 and fibronectin (Fig. S7d,e). We also attempted to quantify the knockdown effects on the cancer cell invasion in the 3D collagen matrix by counting cancer cells attached to invading fibroblasts on fluorescent images for one cross section per spheroid. The Panc-1 cell adhesion to the fibroblast protrusions in the collagen matrix was significantly blocked by both the integrin α5 and the fibronectin knockdown (*p* < 0.05 and *p* < 0.01, respectively) (Fig. 6b,c and Video 7). These results indicate that the integrin α5β1/fibronectin interaction is required not only for the heterotypic cell adhesion in both 2D and 3D conditions but also for the chimeric spheroid formation in the micro-fabricated vessels. Moreover, poor interaction between cancer cells and fibroblasts in the 3D collagen matrix was observed when the Panc-1/WI-38 chimeric spheroids without siRNA treatment were treated with the anti-integrin-α5β1 or the anti-fibronectin antibody (*p* < 0.05 and *p* = 0.066, respectively) (Fig. 6d,e). These antibody treatments also appeared to suppress the invasion of fibroblast protrusions.

### Localization of cell adhesion molecules

**Figure 7:**
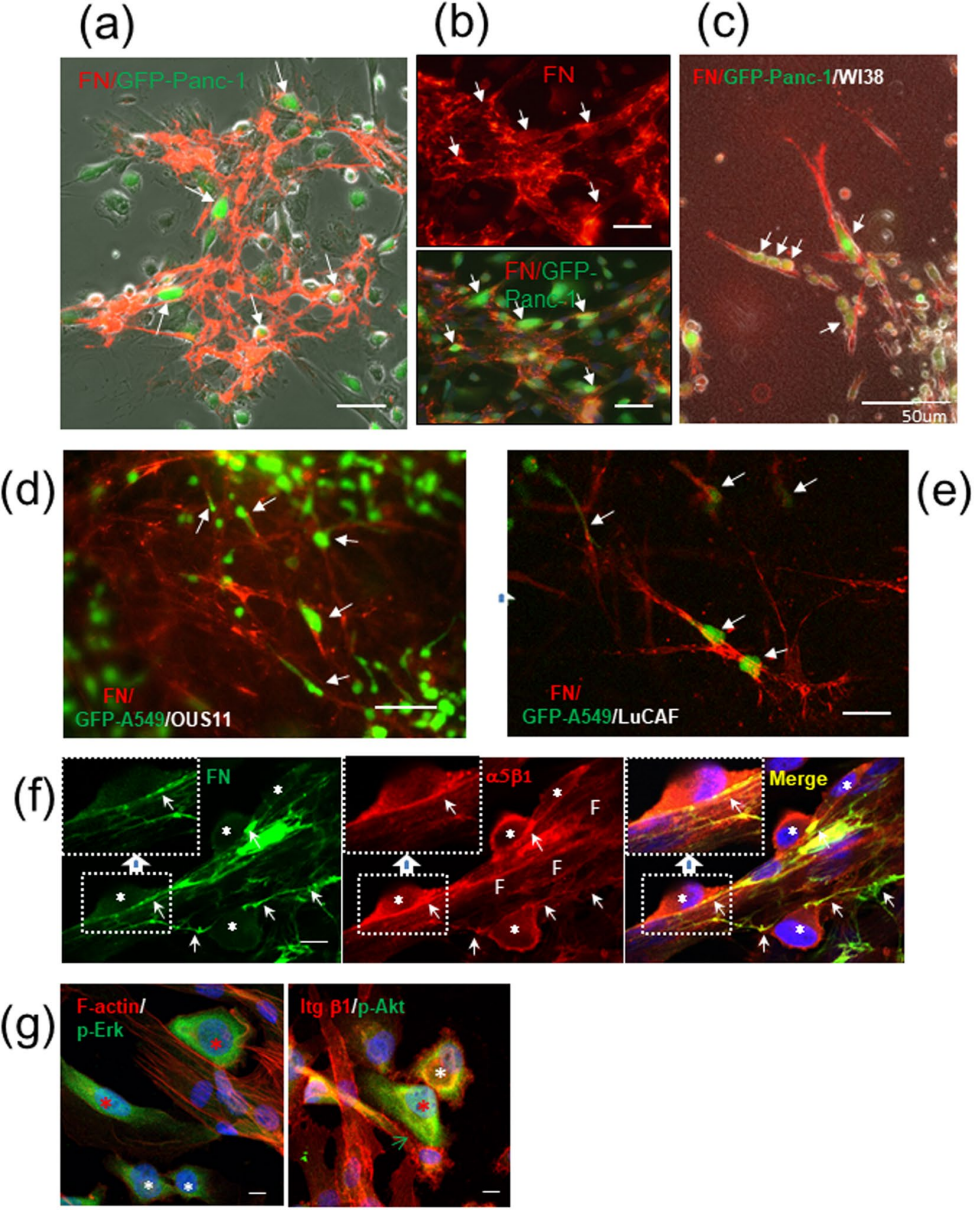
Cancer cells adhere to fibroblasts with fibronectin (FN) deposits and become activated. (**a**,**b**) Immunofluorescent (IF) staining for FN (red) on WI-38 cells in 2D co-cultures. Arrows point Panc-1 cells (green) attaching to fibroblasts. Scale bars, 50 μm. (**a**) A phase-contrast image with FN signals. (**b**) IF image of FN fibrils (upper panel) and its merged image with GFP (Panc-1) (lower panel). Scale bars, 100 μm. (**c**–**e**) Cancer cell (green) invasion in contact with fibroblast protrusions with FN deposits (red) in 3D collagen gel: GFP-Panc-1/WI-38 (**c**), GFP-A549/OUS-11 (**d**), and GFP-A549/LuCAF (**e**). (**f**) Co-localization of integrin α5β1 (red) with FN fibrils (green) at non-labeled Panc-1/WI-38 contact sites (arrows) in 2D co-culture. Blue, DAPI stain; asterisks, Panc-1 cells; F, fibroblasts. Insets, magnified views of the indicated boxed areas. Scale bars, 10 μm. (**g**) IF staining of Panc-1/WI-38 co-culture for phosphorylated Erk (p-Erk, green) and F-actin (red) in the left panel and for phosphorylated Akt (p-Akt, green) and integrin β1 (red) in the right panel. Red asterisks, fibroblast-bound, activated cancer cells; white asterisks, unbound cancer cells; green arrow, positive signals in cancer cells for integrin β1 at the heterotypic contact site. When the p-Erk and p-Akt signals were quantitated using the NIH image J software, the relative intensity for the cellular p-Erk signal was 100 and 70 (85 in average) in the red asterisks and 58 and 35 (47 in average) in the white ones, while that for p-Akt was 100 (red asterisk) and 64 (white asterisk).

The mechanism for the heterotypic cell-cell adhesion was further verified by immunocytochemistry. Immunofluorescent staining of 2D co-cultures for fibronectin revealed that abundant fibronectin fibrils, to which Panc-1 cells appeared to adhere, were assembled on WI-38 cells (Fig. 7a,b). Similar or rather stronger signals for the deposited fibronectin fibrils were found on LuCAFs, Ctr-NLFs and OUS-11 cells in 2D cultures (Fig. S1c). The assembly of fibronectin fibrils on fibroblasts was further confirmed in the 3D co-cultures of the Panc-1/WI-38 (Fig. 7c), A549/OUS-11 (Fig. 7d) and A549/LuCAF (Fig. 7e). Together with the inhibition data shown in Figs 4–6, these immunostaining data indicate that cancer cells attach to and migrate on fibronectin fibrils deposited on fibroblasts. Notably, A549 cells extended long invasive protrusions along the fibronectin-rich fibroblast strings (Fig. 7d,e).

We also analyzed the localization of integrin molecules in the 2D Panc-1/WI-38 co-cultures. Integrin α5β1 was co-localized with fibronectin fibrils at the Panc-1/WI-38 contact sites (Fig. 7f). It is known that the polymerized fibronectin has stronger cell adhesion activity than the monomer^30^. Therefore, it was expected that the cancer cells bound to the fibrillar fibronectin might have stronger intracellular signaling than those bound to the monomer fibronectin or unbound cells. Immunofluorescent staining for phospho-Erk1/2 (p-Erk) (Fig. 7g, left panel) and phospho-Akt (p-Akt) (Fig. 7g, right panel) indicated that these signaling mediators were indeed activated in the cancer cells bound to fibroblasts.

All the results described above support the mechanism that Panc-1 and A549 cancer cells bind to fibroblasts through the integrin α5β1/fibronectin interaction and migrate on the fibroblast protrusions in the collagen matrix. To examine the possible involvement of the E-cadherin/N-cadherin interaction in our heterotypic cell-cell adhesion models, we analyzed the expression of E-cadherin and N-cadherin in the 2D co-cultures of Panc-1 or A549 cancer cells with WI-38 fibroblasts. In immunoblotting analysis, E-cadherin expression was negligible in Panc-1 cells (<0.4%) and far lower in A549 cells (<17%) than that in MCF-7 cells, but the N-cadherin expression levels were comparable among the three cancer cell lines and WI-38 fibroblast (Fig. S8a). Immunofluorescent staining was unable to detect any clear E-cadherin localization in the A549/WI-38 contact sites (Fig. S8b). Even in the co-cultures of the high E-cadherin-expressing cell lines (MCF-7 and A431) with WI-38 cells, localization of E-cadherin at their heterotypic contact sites was not evident in our experimental conditions (Fig. S3a–d). In the case of the MCF-7/WI-38 combination, localization of integrin α5β1 in MCF-7 cells was detected at their contact sites with fibronectin-binding WI-38 cells but less evidently compared with the case of Panc-1 cells (Fig. S8c, see also Fig. 7f). This suggested that the integrin α5β1/fibronectin interaction might also be involved in the weak adhesion of MCF-7 cells to WI-38 cells (Fig. S3a).

### Interaction between integrin α5β1 and fibronectin in invasive adenocarcinomas

**Figure 8:**
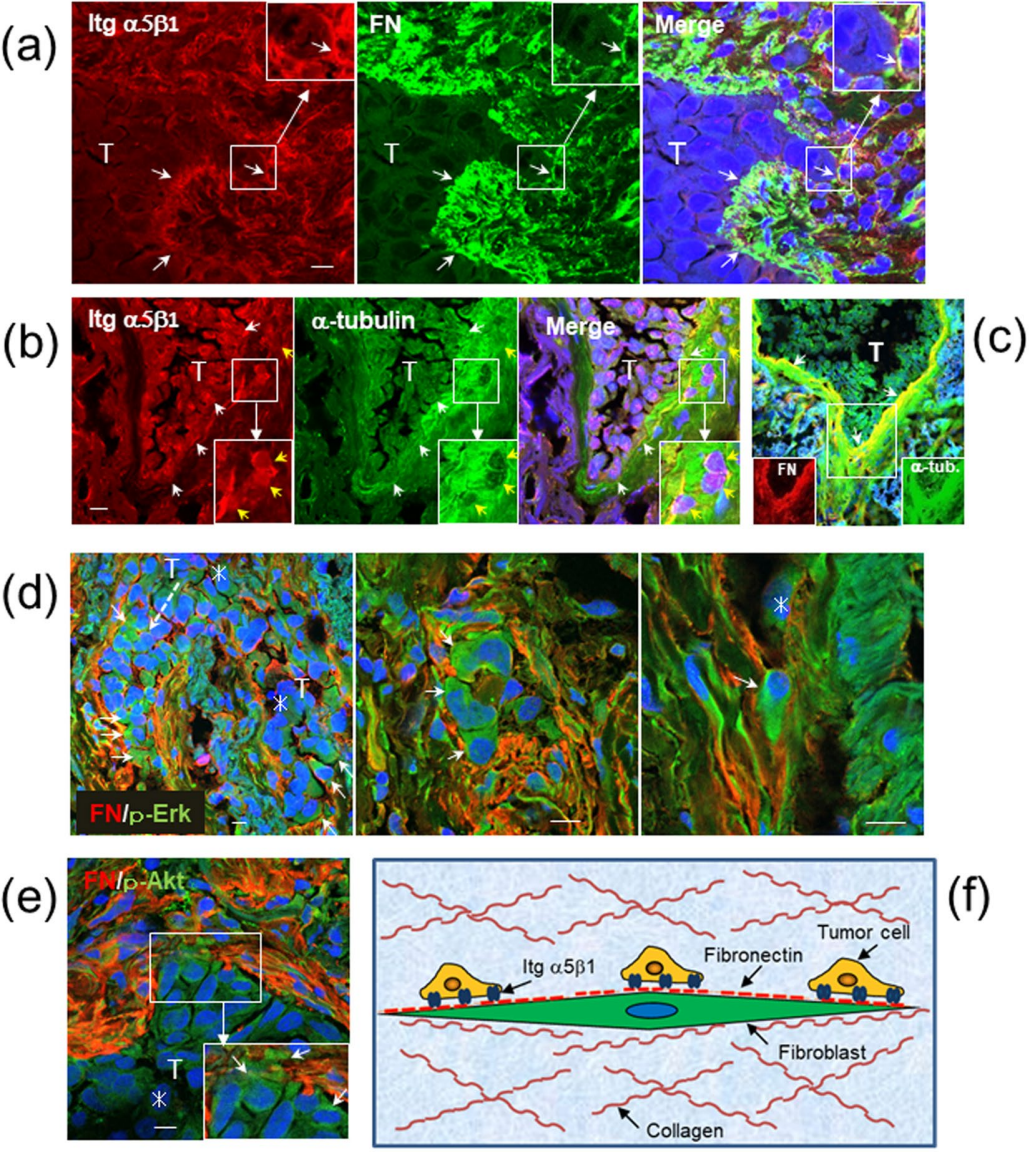
Lung cancer cells at invasion fronts express integrin α5β1, interact with stromal fibronectin (FN) fibrils, and become activated. Adenocarcinomas from patients 1 (**a**,**e**), 2 (**b**,**c**) and 3 (**d**) were subjected to immunofluorescent (IF) staining and then confocal microscopy. T, cluster of cancer cells. Small arrows point co-localization of two kinds of IF signals at invasion fronts in (**a**–**c**), or positive signals for activated cancer cells (**d**,**e**). Insets, magnified views of the indicated boxed areas. Scale bars, 10 μm. (**a**) Integrin (Itg) α5β1 (red), FN (green) and DAPI (blue). (**b**) Itg α5β1 (red), α-tubulin (green) and DAPI (blue). Yellow arrows indicate cancer cells having singly invaded into stroma. (**c**) A merged image of FN (red), α-tubulin (green) and DAPI (blue) in a serial section of (**b**). (**d**) FN (red), p-Erk (green) and DAPI (blue). (**e**) FN (red), p-Akt (green) and DAPI (blue). Asterisks indicate inner cancer cells. Note that cancer cells invading deeply into FN-rich stroma (arrows) show stronger positive signals of p-Erk and p-Akt than inner cancer cells (asterisks) in (**d**,**e**). (**f**) A schematic model of cancer cell migration on fibroblast protrusion via integrin α5β1/fibronectin interaction.

The possible role of the integrin α5β1/fibronectin interaction in human cancer invasion was investigated by immunohistochemistry of human lung adenocarcinoma tissues. In at least 10 cases of invasive lung adenocarcinomas tested, invasion fronts of cancer cells were surrounded by the stroma rich in fibronectin fibrils. Double immunofluorescent staining revealed co-localization of integrin α5β1 on cancer cells with the stromal fibronectin at the invasion fronts (Fig. 8a, insets). In another case of adenocarcinoma, the stromal tissue directly surrounding a cluster of integrin-α5β1-positive cancer cells was strongly stained for α-tubulin (Fig. 8b). This confocal image also shows a few cancer cells which have singly invaded into the adjacent α-tubulin-rich stroma (Fig. 8b, insets). These invading cells were also positive for integrin α5β1. At the invasion fronts of the same tissue, the strong α-tubulin signals were co-localized with strong fibronectin signals (Fig. 8c). Because microtubules construct various cell structures including string-like protrusions of fibroblasts, the dense fibronectin fibrils in the tumor stroma are likely to be mainly cell-associated fibronectin fibrils rather than the fibronectin deposits in the ECM. This possibility was also supported by close localization of the immunofluorescent signals for fibronectin and the mesenchymal cell marker vimentin in the lung cancer tissues (Fig. S8d,e). Some cancer cells appeared to directly attach to the vimentin-positive CAF-like cells.

The cancer cells collectively or singly invading into dense fibronectin fibrils showed stronger positive signals for the activated Erk (p-Erk) (Fig. 8d) and the activated Akt (p-Akt) compared with inner cancer cells in fibronectin-less environments (Fig. 8e). These data also support the mechanism that the binding of cancer cells to fibroblasts via the integrin α5β1/fibronectin interaction promotes cancer cell invasion.

## Discussion

How do cancer cells migrate through the 3D matrix *in vivo*? This has long been investigated as one of the principle subjects in cancer biology. In the 2D culture conditions, cancer cells attach to a suitable substrate via cell-surface adhesion molecules and spread by constructing a new actin cytoskeleton. These cellular changes provide the contractile force for the cells to migrate. How is this scenario attained in the 3D matrix? Although the cell migration mechanism is much more complex in the 3D conditions than the 2D ones, an appropriate cell adhesion substrate is essential for the cells to migrate in both 2D and 3D conditions. The present study using the 2D and 3D co-culture models demonstrates a novel tumor invasion mechanism by which invasive cancer cells migrate on the long strings of fibroblast protrusions in the 3D collagen matrix as if trains run on a railroad track (Fig. 8f). The cancer cell migration was mediated by their adhesion to the fibrillary fibronectin assembled on the surface of fibroblasts. The integrin-mediated binding to the fibronectin is expected to supply a sufficient migratory force via the integrin signaling to the cancer cells. This was supported by the results from the immunostaining experiments of the 2D co-cultures and lung cancer tissues for p-Erk and p-Akt. The extended or elongate morphology of cancer cells in contact with fibroblasts in the 3D matrix also suggested that they acquired enough tension for their migration from the heterotypic cell-cell adhesion.

The heterogeneous cell-cell interaction has been reported by a relatively small number of studies. Gaggioli *et al*.^22^ showed a 3D tumor invasion model in which fibroblasts lead collective cell migration of squamous carcinoma cells by making tracks for the following cells. In a similar model, Labernadie *et al*.^21^ recently demonstrated that fibroblasts lead collective migration of A431 cells by pulling the cancer cells via the E-cadherin/N-cadherin junction. The intercellular junction via E-cadherin/N-cadherin heterophilic interaction has been reported by other groups^31^. In the present study, we also showed fibroblast-dependent cancer cell invasion in the 3D matrix. In our model, however, fibroblasts function as a track for running vehicles rather than the traction vehicles themselves. Moreover, our results with function-blocking antibodies and siRNAs for gene silencing clearly indicated that the principal molecules mediating the heterotypic cell-cell adhesion were integrin α5β1 on cancer cells and fibronectin assembled on the surface of fibroblasts. However, our results do not exclude the possible involvement of other additional cell adhesion molecules in the intercellular adhesion. E-cadherin is down-regulated at later stages of cancer progression, while N-cadherin is reciprocally up-regulated^27,28^. N-cadherin is thought to be an EMT marker, and its expression is associated with the invasive activity of cancer cells^28^. In the present study, Panc-1 and A549 cell lines, as well as fibroblast cell lines, expressed N-cadherin at high levels. N-cadherin and other cell adhesion molecules such as integrins α9β1, α4β1 and αvβ3 for fibronectin^16,32^, cadherin-23^24^, nectins/afadin^21,33^, and integrin α4β1/V-CAM^34^ may also support the heterotypic cell-cell adhesion. However, the stable cell adhesion mechanisms such as the adherence and tight junctions seem unsuitable for our cancer invasion model. It should be emphasized that cancer cells which have lost E-cadherin still have a strong capacity to bind to fibroblasts. It is expected that cancer cells having low affinity for fibroblasts would have poor invasive activity in the 3D co-culture model. In preliminary experiments, A431 cells also showed the fibroblast-dependent invasion in the 3D invasion assay but at a very low speed compared with Panc-1 and A549 cells.

Fibronectin is a major ECM component produced by stromal fibroblasts^16^ and essential for the mesodermal cell migration and mesoderm formation in the development of mouse^35^. However, it has not been thought to be a molecule that mediates the intercellular adhesion. Soluble fibronectin is assembled on cell surface to form polymerized fibronectin fibrils^16^. The major cell receptor for fibronectin and its assembly is integrin α5β1^36,37^. The binding of soluble fibronectin to the integrin causes its conformational change from the compact form to the extended form, thus exposing the cell-binding sites and fibronectin-binding sites^16^. Polymerized fibronectin exerts much higher cell adhesion activity than the soluble one^30^. In addition to integrin α5β1, integrin αvβ3, α9β1, α4β1 and syndecan-4 are thought to interact with fibronectin^16^. Among them, integrin α9β1 specifically recognizes an EIIIA-containing isoform of fibronectin^32^. There are numerous studies showing that the integrin/fibronectin interaction is involved in cancer progression^17^. Fibronectin is over-expressed in primary and metastatic cancer tissues, and it is associated with poor survival of the cancer patients^18,38^. Similarly, high expression of integrin α5β1 correlates with poor prognosis in cancer patients^39,40^. The integrin α5β1-mediated adhesion to fibrillary fibronectin facilitates cancer cell invasion by activating FAK, Erk, Akt and other signal mediators and hence remodeling cytoskeleton^41–43^.

Very recently, three groups have reported important roles of fibronectin matrices in CAF-guided cancer cell migration in 2D cultures. CAFs lead invasion of CT26 mouse intestinal cancer cells by assembling fibronectin via integrin αvβ3^19^. GAL33 squamous carcinoma cells collectively migrate via integrins αvβ6 and α9β1, rather than α5β1, on 2D cell-free fibronectin-rich matrices^18^. Moreover, Erdogan *et al*.^20^ found that CAFs promote directional migration of DU145 prostate carcinoma cells by organizing aligned fibronectin matrix. In this case, CAFs utilized integrin α5β1 to organize the fibronectin matrix, whereas cancer cells did αv integrins as the fibronectin receptors. Consistent with these studies, we showed important functions of CAF-derived fibronectin in cancer cell invasion. In our study, however, cancer cells utilized integrin α5β1 at least as a major receptor to bind to fibronectin fibrils on fibroblasts. Fibronectin receptors may vary depending on the type of cancer cells.

Time-lapse experiments in the present study demonstrated that cancer cells preferentially adhered to the elongate protrusions of fibroblasts through the integrin α5β1/fibronectin interaction and efficiently migrated through the collagen matrix. The fact that cancer cells which left the fibroblast protrusions greatly lost their invasive potential suggested that the cancer cells bound to fibroblasts acquired the migratory force by the integrin-mediated signaling. The collagen gel co-cultures seem to contain soluble fibronectin and its polymers released from fibroblasts. Indeed, the immunofluorescent staining detected fibronectin-rich membrane fragments around the chimeric spheroids (Fig. 7c–e). However, these cell-free fibronectins appeared unsuitable as the substrate that sufficiently supports cancer cell spreading and migration. The fibronectin fibrils anchored to fibroblasts seem to function as the most favorable substrate for cancer cells to migrate through the collagen matrix. Fibronectin is known to interact with collagen and other ECM components^16^. However, it is unclear how fibronectin exists in the tumor stroma. As reported in other types of cancers^18,20^, fibrillary fibronectin was densely detected at the invasion fronts of cancer cells in lung cancer tissues. Such accumulation of fibronectin fibrils could be produced by CAFs, at least in parts, as a result from the stimulation by invading cancer cells. The co-localization of α-tubulin with the fibronectin matrix suggests that the fibrillary fibronectin is associated with the filamentous protrusion of CAFs. It is supposed that such fibronectin fibrils serve for a good substrate of cancer cell growth and invasion *in vivo*.

It is thought that there are differences between resting and activated fibroblasts, or between normal fibroblasts and CAFs, in many aspects, e.g. activities of proliferation, migration and ECM production^4,8,20^. In our experimental model, the fibroblast-dependent cancer cell migration was similarly found in all of four types of fibroblasts though their invasion-supporting activity differed among the cell types. The difference in the activity between the lung CAFs (LuCAFs) and their normal counterparts (Ctr-NLFs) was, if any, not significant. Considering the diversity in the origins and phenotypes of fibroblasts in cancer tissues^3–5^, the functional difference should be investigated in more detail using many different pairs of fibroblasts in future studies. We previously found that TGF-β, which is expressed in Panc-1 cells^29^, enhances the expression of fibronectin, angiomodulin and α-SMA in WI-38 cells and human dermal fibroblasts^44^. In Panc-1 and A549 cells, TGF-β down-regulates E-cadherin, up-regulates EMT markers such as vimentin, laminin-γ2 and MMPs, and enhances the activities of these cells to adhere to fibronectin and to migrate^45^. Thus, the direct interaction between fibroblasts and cancer cells as well as the indirect one via TGF-β and other soluble factors are expected to affect the fibronectin assembly in fibroblasts and the invasive activities of both types of cells.

In conclusion, we propose a new tumor invasion mechanism by which cancer cells bind to fibroblast-associated fibrillary fibronectin via integrin α5β1, leading to the activation of the integrin signaling and to the enhanced cancer cell migration along the network of fibroblast protrusions. It is expected that our results provide a clue to clarify the central mechanism by which cancer cells invade human tissues.

## Materials and Methods

### Antibodies and reagents

The sources and types of primary antibodies used in this study are listed in Table S1. In addition, anti-mouse-IgG and anti-rabbit-IgG goat polyclonal antibodies labeled with Cy3 or FITC were purchased from Vector (Burlingame, CA). For double immunofluorescent staining with mouse antibodies, the anti-fibronectin monoclonal antibody FN12-8 was labeled with FITC using Dojindo Ab-10 Rapid Fluorescein Labeling Kit (Kumamoto, Japan). The RGD peptide (Gly-Arg-Gly-Asp-Ser-Pro) and the control RGE peptide (Gly-Arg-Gly-Glu-Ser-Pro) were purchased from Takara (Shiga, Japan).

### Cell lines and culture condition

All human cancer cell lines used in this study are listed in Table S2^46^. GFP-labeled Panc-1 and A549 cell lines were previously established by introducing the pAcGFP-N3 vector from Clontech (Mountain view, CA) into these cell lines^29^. Human fetal lung fibroblast line WI-38 and adult lung fibroblast line OUS-11 (JCRB1034), which had been established from a non-neoplastic tissue of a lung cancer by Dr. M. Namba (Okayama University, Japan), were provided from Japanese Collection of Research and Bioresources (JCRB, Tokyo, Japan). Primary cultures of human lung cancer-associated fibroblasts (Lu-CAFs) and control normal fibroblasts (Ctr-NLFs) were prepared by the method of Holz *et al*.^47^ from a tumor tissue and a normal parenchymal tissue distal from any tumors, respectively, both which were obtained by lung resection for a localized lung adenocarcinoma in Juntendo University Hospital. The Institutional Review Board at the Juntendo University School of Medicine approved the procedures. The patient provided written, informed consent (No. 20042). Experimental procedures were performed according to the institutional guidelines and those of the 1995 declaration of Helsinki. The above cell lines were used within 5 continuous passages. All these cell lines were maintained in DMEM/F12 medium (Invitrogen, Carlsbad, CA) supplemented with 10% fetal bovine serum (FBS) at 37 °C in a humidified atmosphere of 5% CO_2_ and 95% air.

### Heterotypic cell adhesion assays in two-dimensional (2D) culture

In quantitative cell adhesion assays, WI-38 cells (2 × 10^4^ cells/well) were pre-incubated for 3 days on 24-well culture plates. GFP-labeled cancer cells (2 × 10^4^ cells) were inoculated into each well of the WI-38 culture and further incubated for 2 h. Phase-contrast images with GFP fluorescence signals were obtained from a center position of each well in an inverted CKX41 microscope equipped with a fluorescence illumination unit (Olympus, Tokyo). Of cancer cells in contact with fibroblasts, the percentage of elongate cancer cells was determined from representative images with similar cell distribution. The elongate cancer cells were defined as the cells having the length to width ratios of over 1.5. In alternative assays, cancer cells were inoculated on confluent WI-38 cells and incubated for 1 h. After washing out unattached and loosely attached cancer cells, the total number of cancer cells remaining on the fibroblast layer was determined using the NIH imageJ software (Fiji). Time-lapse experiments were carried out using sub-confluent co-cultures on 35-mm culture dishes with 12-mm glass bottom (AGC Techno Glass, Shizuoka). The cultures were incubated in the Keyence digital fluorescence microscope BZ-9000 equipped with a CO_2_ incubator (Osaka, Japan), taking phase-contrast images with GFP signals at 5-min intervals.

### Cancer cell invasion assay in three-dimensional (3D) collagen gel

EZSPHERE micro-fabricated 96-well plate (AGC Techno Glass) has about 90 small pores in each well and hence allows to easily make many homogeneous spheroids of cells^48^. Using this plate, chimeric spheroids with 120–150 μm in diameter were prepared by incubating 1.5 × 10^4^ cells/well each of GFP-labeled cancer cells and fibroblasts for 20 h. The spheroids were recovered into a 1.5-ml tube from each well and precipitated by centrifugation at 800 rpm for 2 min. For collagen gel culture, cold 1.9 mg/ml collagen solution containing × 1 DMEM/F12 and 10% FBS was prepared from 0.3% bovine dermis native collagen (Koken Atelo Cell IAC-30, Tokyo). Seventy μl of the unsolidified collagen/medium solution was placed on the 12-mm glass bottom of 35-mm culture dishes, spread and solidified at 37 °C for at least 30 min. The spheroid precipitate was suspended in 100 μl of the ice-cold collagen/medium solution, and its 50-μl aliquot was spread on the lower collagen gel and solidified. Two ml of the complete medium was gently added to each dish, and the 3D collagen gel culture was incubated at 37 °C in a CO_2_ incubation chamber of the Keyence digital fluorescence microscope BZ-9000. Time-lapse images were taken every 30 min for one representative spheroid after pre-incubation for 1 h unless otherwise noted. To quantitate the fibroblast-dependent cancer cell invasion, cancer cells bound to invading fibroblasts were counted on representative fluorescent images with one cross section per spheroid from triplicated dishes.

### Inhibition of cell adhesion by function-blocking antibodies

To block cell adhesion proteins in 2D co-cultures, GFP-labeled Panc-1 or A549 cells were pre-incubated with 10 μg/ml of normal mouse IgG, 1 μg/ml of anti-E-cadherin antibody, or anti-integrin antibodies at a 100-fold dilution in the standard medium at room temperature (RT) for 30 min. After the treatment, the cells were transferred to the WI-38 cell culture and incubated for 2 h, followed by the cell adhesion assay as described above. The concentrations of IgG and antibodies were diluted 1.5-fold in the final assay medium. In the case of the anti-fibronectin antibody FN12-8, WI-38 cells were pre-treated with 10 μg/ml of the control mouse IgG or FN12-8 at RT for 2 h. GFP-labeled cancer cells were then applied to the WI-38 cell culture. The concentrations of the test samples were diluted two-fold in the final assay medium. In the case of 3D collagen co-cultures, control IgG or FN12-8 was included at 10 μg/ml in the upper collagen gel and the culture medium.

### siRNAs and transfection

Knockdown experiments of integrin α5 and fibronectin were performed using pools of 4 siRNAs and a negative control RNA (Dhamacon SMARTpool; GE Healthcare) (Table S3). These RNAs were transfected to Panc-1 or A549 cancer cells or WI-38 fibroblasts using Lipofectamine RNAiMAX (Invitrogen). Two or three days after the transfection, these cells were used to see the knockdown effects.

### Immunochemical analyses

Immunofluorescent staining was performed using the specific antibodies listed in Table S1, according to the standard protocol. Briefly, cultured cells or 5-μm frozen sections of human cancer tissues were fixed in 10% formalin/PBS and blocked with 3% (w/v) bovine serum albumin (BSA)/PBS. These samples were treated with the primary antibody, such as FN12-8 (fibronectin) at x 200 dilution and #1969 (integrin α5β1) at x100 dilution at RT for 2 h or at 4 °C overnight (Table S1) and then with the secondary antibody conjugated with Cy3 (red), Alexa Fluor 488 (green) or FITC (green) (x200 dilution) at RT for 1 h. For intracellular proteins, fixed samples were permeabilized with 0.1% (v/v) Triton X-100 in PBS for 15 min. In the case of collagen gel cultures, stained samples were washed with at least 5 changes of PBS for a few days. Fluorescent images were obtained under a Carl Zeiss LSM 710 laser scanning microscope with the software ZEN (Jena, Germany) or the Keyence digital fluorescence microscope BZ-9000. Human cancer specimens were obtained from patients who received surgery at the Kanagawa Cancer Center (KCC) Hospital (Kanagawa, Japan) between 2006 and 2009 and provided by Human Cancer Tissue Bank of KCC. Written informed consent was obtained from each patient in KCC Hospital. The study protocol was approved by the Ethical Committees of both KCC and Kihara Institute for Biological Research, Yokohama City University, and experimental procedures were performed according to the institutional guidelines and those of the 1995 declaration of Helsinki.

### SDS-PAGE and immunoblotting analysis (Western blotting)

SDS-PAGE was performed on Bio-Rad Mini-Protean gels (4–15% gel for fibronectin and Any kD gels for other proteins). In the cases of knockdown experiments, confluent culture in each well of 24-well plates were lysed with 50 μl of the Laemmli’s SDS sample buffer with 2-mercaptoethanol, and 10 μl of the cell lysates was applied on each lane. In other cases, confluent cultures in 6-cm culture dishes were dissolved in 0.5 ml of a lysis buffer containing 1% (v/v) Triton X-100, extracted by centrifuging at 15,000 rpm for 30 min, and analyzed for protein concentration with the Bio-Rad DC Protein Assay kit. After SDS treatment, the samples were applied to SDS-PAGE at constant protein loading. The separated proteins were transferred onto PVDF membranes and subjected to the standard immunoblotting. As primary antibodies, anti-integrin-α5 polyclonal antibody (Santa Cruz), anti-fibronectin monoclonal antibody FN9-1, and other specific antibodies were used at x500 or x1000 dilution (Table S1). The immunosignals were detected with the Amersham Enhanced Chemiluminescence (ECL) Detection Reagent in the ImageQuant LAS 4000 analyzer (GE Healthcare). Band volume was measured with the software ImageQuant TL.

### Statistical analysis

Statistical significance was evaluated with an unpaired, two-tailed Student’s T test. A *p* value < 0.05 was considered significant. Unless otherwise noted, all statistic data shown are the means ± S.D. in triplicate cultures. When representative images were shown, they represent at least three samples.

## Electronic supplementary material

Supplementary information

Video 1

Video 2

Video 3

Video 4

Video 5

Video 6

Video 7

## Data Availability

All data supporting the findings in this study are available from the corresponding author on reasonable request.
