# Supplementary material for: Cancer cell migration on elongate protrusions of fibroblasts in collagen matrix
Source: Sci Rep. 2019 Jan 22;9:292. doi: 10.1038/s41598-018-36646-z (PMC6342997; doi:10.1038/s41598-018-36646-z)
Supplement: Supplementary file 1 — Supplementary information [file 41598_2018_36646_MOESM1_ESM.pdf]

SUPPLEMENTARY INFORMATION

# Cancer cell migration on elongate protrusions of fibroblasts in collagen matrix

**[1,2*]Kaoru Miyazaki, [2¤]Jun Oyanagi, [3]Daisuke Hoshino, [4]Shinsaku Togo, [5]Hiromichi Kumagai, [1]Yohei Miyagi**

[1]Molecular Pathology and Genetics Division and [3]Cancer Cell Biology Division, Kanagawa Cancer Center Research Institute, 2-3-2 Nakao, Asahi-ku, Yokohama 241-8515, Japan; [2]Division of Cell Biology, Kihara Institute for Biological Research, Yokohama City University, 641-12 Maioka-cho, Totsuka-ku, Yokohama 244-0813, Japan; [4]Division of Respiratory Medicine, Juntendo University of Medicine, 3-1-3 Hongo, Bunkyo-Ku, Tokyo113-8431, Japan; [5]Kumagai Fellow laboratory, Innovative Technology Research Center, Technology General Division, AGC Inc., 1150 Hazawa-cho, Kanagawa-ku, Yokohama 221-8515, Japan.

## Video Information

**Video 1.** Panc-1 cells quickly adhere, spread and migrate on semi-confluent WI-38 fibroblasts in 2D co-culture. WI-38 cells were pre-cultured for 3 days to reach semi-confluence in a 35-mm culture dish. GFP-labeled Panc-1 cells (green) were inoculated on the fibroblast culture and the dynamics of co-culture was recorded at 5-min intervals as phase-contrast time-lapse images with GFP signals. Arrows trace 2 migrating cancer cells extending filopodia-like pseudopodia. See also Figs. S2a. Scale bars, 50 μm.

**Video 2.** A549 cells rapidly migrate in contact with semi-confluent WI-38 fibroblasts in 2D co-culture. GFP-labeled A549 cells (green) were inoculated on a semi-confluent culture of WI-38 cells, and time-lapse images were obtained as described in Video 1. A549 cells more rapidly migrate on the fibroblasts than Panc-1 cells. Arrows trace 2 migrating cancer cells extending lamellipodia-like protrusions. See also Fig. 1c. Scale bars, 50 μm.

**Video 3.** Panc-1 cells migrate on extended protrusions of WI-38 cells in 3D collagen matrix. Chimeric spheroids of GFP-labeled Panc-1 cells (green) and WI-38 fibroblasts (non-colored) were prepared, embedded in collagen gel and pre-incubated at 37 ˚C. The time-lapse experiment was started 20 h after the initial inoculation and recorded at 30 min intervals under a fluorescence microscope. During the pre-incubation, WI-38 fibroblasts sufficiently migrated through the collagen matrix away from the spheroid. As shown by yellow and white arrows, Panc-1 cells quickly migrate on the fibroblast protrusions. See also Figs. 2bcd. Scale bars, 50 μm.

**Video 4**. Both A549 cells and OUS-11 fibroblasts actively invade the 3D collagen matrix compared with the combination of Panc-1 and WI-38. Chimeric spheroids of GFP-labeled A549 cells (green) and OUS-11 fibroblasts (non-colored) were subjected to the invasion assay in 3D collagen matrix as described in Video 3. Time-lapse images of a representative spheroid were obtained at 30-min intervals after 22-h pre-incubation. A white arrow traces one cancer cell migrating on fibroblast strings changing four fibroblasts, while two black arrows point cancer cells showing reciprocating movement on one fibroblast string. It is also noted that OUS-11 fibroblasts more actively migrate than WI-38 cells. See also Fig. 3bcd. Scale bars, 100 μm.

**Video 5**. Invasion of A549 cells and lung CAFs in 3D collagen matrix. Chimeric spheroids of GFP-labeled A549 cells (green) and CAFs (non-colored) were subjected to the invasion assay in 3D collagen matrix as described in Video 3. Time-lapse images of a representative spheroid were obtained at 30-min intervals after 22-h incubation. It is noted that the lung CAFs more actively migrate than WI-38 and OUs-11 fibroblasts, releasing fragments of invasive protrusions. See also Fig. 3e. Scale bars, 50 μm.

**Video 6**. Function-blocking antibody against integrin α5β1 blocks adhesion of Panc-1 cells to WI-38 fibroblasts in 2D culture. GFP-labeled Panc-1 cells were pretreated with the antibody and inoculated on semi-confluent culture of WI-38 cells as described in Fig.

4b, and the time-lapse images were obtained as described in Video 1. Compare with Video 1. Scale bars, 50 μm.

**Video 7.** Depletion of fibronectin (FN) in WI-38 fibroblasts with specific siRNAs suppresses invasion of Panc-1 cells in 3D collagen gel. Chimeric spheroids of GFP-labeled Panc-1 cells (green) and fibronectin-depleted WI-38 fibroblasts were embedded in collagen gel. Time-lapse images of one representative spheroid were recorded after 22-h pre-incubation. Other experimental conditions are described in Fig. 6. Note that only a few cancer cells migrate on fibroblast protrusions. Compare with Video 3. Scale bar, 50 μm.

## Supplementary Tables

**Table S1. Antibodies used in this study**

| Human antigen | Clones | Types | Usage | Makers |
|---|---|---|---|---|
| Integrin α3 | P1B5 | mMAb | FB | Merck |
| Integrin α5 | P1D6 | mMAb | FB, IH | Merck |
| Integrin α5β1 | #1969 | mMAb | FB, IH | Merck |
| Integrin αv | #1098 | mMAb | FB | Merck |
| Integrin β1 | #2253Z | mMAb | FB, IH | Merck |
| Integrin α4 | 2B4 | mMAb | FB | R&D |
| Integrin α6 | GoH3 | mMAb | FB | PM |
| Integrin α5 | | rabbit PoAb | IB | SC |
| Fibronectin | | rabbit PoAb | IB | SC |
| Fibronectin | FN12-8 | mMAb | FB, IH | Takara |
| Fibronectin | FN9-1 | mMAb | IH, IB | Takara |
| E-cadherin | SHE78-7 | mMAb | FB | Takara |
| E-cadherin | | mMAb | IH | BD |
| E-cadherin | 24E10 | rabbit MAb | IH | CS |
| N-cadherin | | mMAb | IH | BD |
| p-Akt | D9E | rabbit MAb | IH | CS |
| p-Erk1/2 | #4370 | rabbit MAb | IH | CS |
| GAPDH | | HRP-mMAb | IB | Wako |
| αSMA | | mMAb | IH, IB | Sigma |
| α-tubulin | | mMAb | IH | Merck |
| Vimentin | | rabbit FITC-MAb | IH | TF |

Abbreviations: MAb, monoclonal antibody; mMAb, mouse MAb; PoAb, polyclonal antibody; FB, function blocking; IH, immunohistochemistry; IB, immunoblotting. Makers: Merck (Darmstadt, Germany); R&D (Minneapolis, MN, USA); PM, PharMingen (San Diego, CA, USA); SC, Santa Cruz (Dallas, TX, USA); Takara (Shiga, Japan); Becton Dickinson (Franklin Lakes, NJ, USA); CS, Cell Signaling (Danvers, MA, USA); Wako (Osaka, Japan); Sigma (St Louis, Mo, USA); TF, ThermoFisher (Waltham, MA, USA).

**Table S2. Human cancer cell lines used in this study**

| Human antigens | Organs | Types | Sources |
|---|---|---|---|
| A549 | lung | adenocarcinoma | JCRB |
| Panc-1 | pancreas | adenocarcinoma | JCRB |
| MKN-45 | stomach | adenocarcinoma | JCRB |
| MMK-74 | stomach | adenocarcinoma | JCRB |
| A431 | skin | epidermoid carcinoma | JCRB |
| MCF-7 | breast | adenocarcinoma | JCRB |
| MIA-PaCa-2 | pancreas | adenocarcinoma | JCRB |
| CaSki | cervix | squamous cell carcinoma | ATCC |
| MDA-MB-231 | breast | adenocarcinoma | ATCC |
| STKM-1 | stomach | adenocarcinoma | Ref. 46 |

JCRB, Japanese Collection of Research and Bioresources (Tokyo, Japan); ATCC, American Type Culture Collection (Manassas, VA, USA).

**Table S3. Sequences of siRNAs for integrin α5 and fibronectin and their control**

| Integrin α5 | Targeting or non-targeting siRNAs |
|---|---|
| | GAACGAGUCAGAAUUUCGA |
| | UCACAUCGCUCUCAACUUC |
| | ACACGUUGCUGACUCCAUU |
| | CAAACGCUCCCUCCCAUAU |
| Fibronectin | GCAGCACAACUUCGAAUUA |
| | GAAAUAGAUGCAACGAUCA |
| | GAGGAAAUCUGCACAACCA |
| | CCUAAAGACUCCAUGAUCU |
| Negative control | UGGUUUACAUGUCGACUAA |

## Supplementary Figure Legends

**Figure S1.** Activation state of four kinds of human fibroblasts: WI-38, OUS-11, LuCAF, and Ctr-NLF. These cell lines were cultured on 60-mm dishes for 3 days and extracted with a lysate buffer containing 1% Triton X-100. Ten μg each of the extract was subjected to immunoblotting for αSMA (left panel) and fibronectin (FN; right panel). (b) & (c) Two-days cultures of the four cell lines on an 8-well chamber slide were subjected to immunofluorescent staining for αSMA (b) and fibronectin (c). The former staining was performed after permeabilization of the cultures. Fluorescent images were obtained under the same conditions for each staining. Scale bars, 50 μm in (b) and 20 μm in (c).

**Figure S2.** Heterotypic adhesion of different kinds of adenocarcinoma cells to WI-38 fibroblasts in 2D culture conditions. (a) & (b) WI-38 fibroblasts were cultured in 35-mm plastic culture dishes to reach semi-confluence (a) or confluence (b). GFP-labeled Panc-1 cells (green) were plated on the fibroblast cultures and incubated for 2 h (a) or 3 h (b), followed by fluorescence microscopy. Scale bars, 100 μm. Cancer cells bound to fibroblasts more markedly stretched their structures in (b) than (a). (c)-(f) The following cancer cells were labeled by Green Fluorescence Live Cell Tracking Kit (AAT Bioquest): MKN-45 gastric carcinoma cells (c), STKM-1 gastric carcinoma cells (d), MIA-PaCa-2 pancreatic carcinoma cells (e), and MDA-MB-231 mammary carcinoma cells (f). They were co-cultured with WI-38 fibroblasts for 1 day. Arrows point cancer cells which adhered to fibroblasts. Insets are the morphology of the respective cancer cells in the single cultures at the same scale. Scale bars, 100 μm.

**Figure S3.** Some types of cancer cells exhibit island structures in 2D co-cultures with WI-38 fibroblasts. (a) & (b) MCF-7 mammary adenocarcinoma; (c) & (d) A431 vulva epidermoid carcinoma; (e) CaSki cervix squamous cell carcinoma; (f) MKN-74 gastric carcinoma. (b) & (d) Immunofluorescent images for E-cadherin of the rectangular regions of (a) and (c), respectively. Arrows point the same heterotypic contact sites in the left and right panels. Scale bars, 100 μm in (a-e) and 200 μm in (f).

**Figure S4**. Preparation of chimeric spheroids and invasion assays in 3D collagen gel. GFP-labeled Panc-1 cells (green) were incubated overnight without (a) or with non-labeled (b-c) or orange fluorescent dye-labeled (d) WI-38 cells in the micro-fabricated plate EZSPHERE. (a) & (b) Phase-contrast images; (c) merged image of bright and fluorescent views; (d) confocal image. Scale bars, 50 μm in (a) and (d); and 20 μm in (b) and (c). Note that Panc-1 cells alone form loose cell aggregates (a), but they form solid spheroids in the presence of WI-38 cells (b-d). (e) A chimeric spheroid of GFP-labeled Panc-1 cells (green) and WI-38 fibroblasts, whose culture also contained WI-38-free aggregates of Panc-1 cells, was incubated for 1 day in the 3D collagen gel as described in Fig. 2b. Scale bar, 100 μm. Note that Panc-1 cells with invasive protrusions (red arrows) are more abundant in the presence of WI-38 cells (yellow circle) than its absence (white circle), while round non-invasive cancer cells (white arrows) vice versa. (f) Invasive activity of OUS-11 fibroblasts appears higher in chimeric spheroids with A549 cells (right panel) than those with Panc-1 cells (left panel). The chimeric spheroids were incubated for 2 days in 3D collagen gel. Scale bars, 100 μm. Arrows point leading

fibroblasts. The number and distance of invading fibroblasts are greater in A549 cells than Panc-1 cells.

**Figure S5.** Function-blocking integrin antibodies against α5, β1, α5β1 and RGD peptide partially block adhesion of Panc-1 cells to WI-38 fibroblasts in 2D conditions. (a) Panc-1 cell adhesion to confluent cultures of WI-38 cells. (b) Panc-1 cell adhesion to semi-confluent cultures of WI-38 cells. (c) Anti-E-cadherin antibody completely blocks homophilic adhesion of MCF-7 breast cancer cells. Experimental conditions are described in Fig. 4. One representative image from triplicate cultures for each test sample is shown. Scale bar, 100 μm.

**Figure S6.** Knockdown effects of integrin α5 in Panc-1 (a, left panel) and A549 (a, right panel) cells and fibronectin (FN) in WI-38 cells (b). (a) Immunoblots for integrin α5 (Itg α5) in Panc-1 (left panel) and A549 (right panel). GAPDH, internal loading control. The band intensity of Itg α5 in the α5-si-treated cells relative to the Ctrl-treated ones was estimated to be 12% at both 10 nM and 40 nM RNAs in Panc-1; and 23% at 10 nM and 20% at 40 nM in A549 cells after calibration for GAPDH. Parentheses, molecular sizes in kDa. (b) Immunoblots for FN in WI-38 cells. The band intensity of FN in the FN-si-treated cells relative to the Ctrl-treated ones was estimated to be 7.1% at 10 nM and 6.0% at 50 nM after calibration for GAPDH. Other experimental conditions are described Fig. 5.

**Figure S7.** Depletion of fibronectin (FN) in WI-38 fibroblasts with specific siRNAs causes impaired deposition of fibrillary FN on cell surface (a) and cell enlargement (b). WI-38 cells were transfected with 10 nM of control RNA (left panels; Ctrl) or a pool of FN-specific RNAs (right panels; FN siRNA) and then incubated for 3 days in a 24-well plate. These cultures were subjected to immunofluorescent staining for FN (red) (a) and morphological examination by a phase-contrast microscope (b). In (a), the two fluorescence images were obtained at the same conditions. Scale bars, 100 μm. Note that the siRNA-transfected cells show extremely reduced FN staining and enlargement of cell structures (b, right panel, arrows) compared with the control cells. Membrane fragments derived from the cell migration activity are seen only in the control cells (b, left panel). (c) Morphological effect of integrin (Itg) α5 knockdown was examined for Panc-1 cells as above. (d) & (e) Effect of knockdown of Itg α5 in Panc-1 (d) or FN in WI-38 cells (e) on size of Panc-1/WI-38 chimeric spheroids. Chimeric spheroids were prepared for the respective knockdown cells, recovered in tubes and placed in collagen gel, followed by measuring the diameter of each spheroid. n = 10 in (d) and 11 in (e).

**Figure S8.** E-Cadherin is poorly expressed in Panc-1 and A549 adenocarcinoma cells and scarcely detected at contact sites between A549 and WI-38 cells. (a) Expression of E-cadherin and N-cadherin in Panc-1, A549 and WI-38 cells as analyzed by immunoblotting. MCF-7 and HeLa were used for positive controls for E-cadherin and N-cadherin, respectively. The extracts of these 5 cell lines were applied to SDS-PAGE gels at 15 or 30 μg protein/lane and then to immunoblotting with the ECL detection for E-cadherin (left upper panels), N-cadherin (right upper panel) and GAPDH as an internal loading control (lower panels). The ECL images for the cadherins were obtained after 15-

sec exposure in the top panel of E-cadherin (short expos.) or 60 sec in the other panels. N-Cadherin was analyzed at a protein loading of 15 μg/lane. Original immunoblots are shown below. Expression level of E-cadherin relative to MCF-7 cells was estimated to be < 0.4% in Panc-1 cells and < 17% in A549 cells. E-cadherin was never detected in WI-38 cells (data not shown). N-cadherin expression levels were comparable among the three cancer cell lines and WI-38 fibroblasts. (b) Immunofluorescent (IF) staining of A549/WI-38 co-culture for E-cadherin and fibronectin (FN). A merged image for E-cadherin (green), FN (red) and nuclei (DAPI, blue) is shown. The E-cadherin image was obtained with the rabbit monoclonal antibody 24E10. Any clear signal for E-cadherin was not detected in their cell contact sites though this antibody strongly stained the cell-cell adhesion junctions of MCF-7 cells. (c) IF images of MCF-7/WI-38 co-culture for FN (green, top panel) and integrin α5β1 (red, center panel). The bottom panel is the merged image. In (b) and (c), arrows point adhesion sites of cancer cells (asterisks) to fibroblasts (F). Scale bars, 10 μm. (d) and (e) IF staining of two lung cancer tissues for vimentin (Vim) (green) and FN (red). Small white arrows, Vim-positive cells; T, tumor cells; yellow arrows, direction of tumor cell invasion. The staining samples of (d) and (e) were close sections of those used in Fig. 8b and Figs. 8ae, respectively. Bars, 10 μm.

# Fig. S1

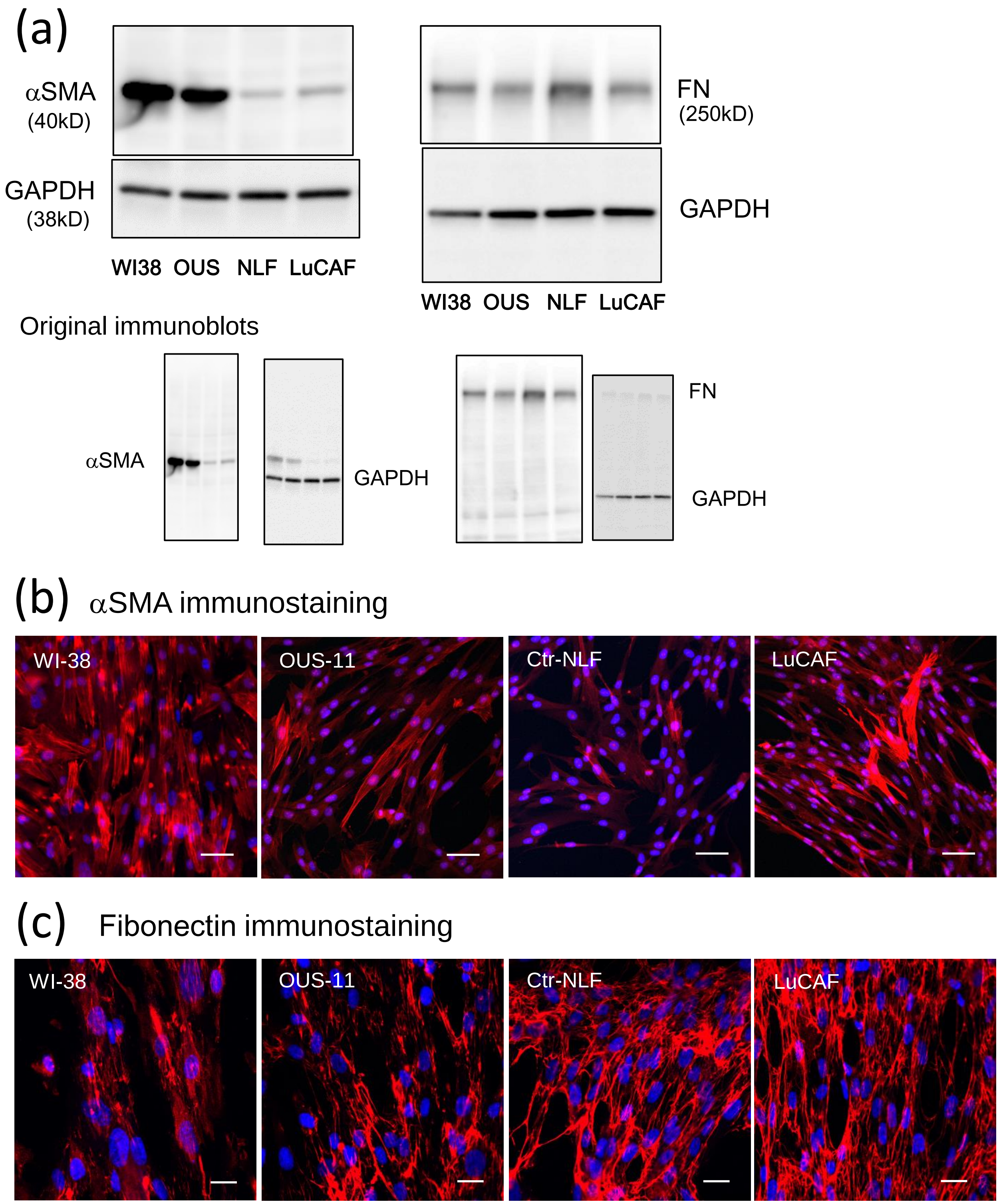
(a)
αSMA
(40kD)
GAPDH
(38kD)
WI38 OUS NLF LuCAF
FN
(250kD)
GAPDH
WI38 OUS NLF LuCAF
Original immunoblots
αSMA
GAPDH
FN
GAPDH
(b) αSMA immunostaining
WI-38
OUS-11
Ctr-NLF
LuCAF
(c) Fibonectin immunostaining
WI-38
OUS-11
Ctr-NLF
LuCAF

# Fig. S2

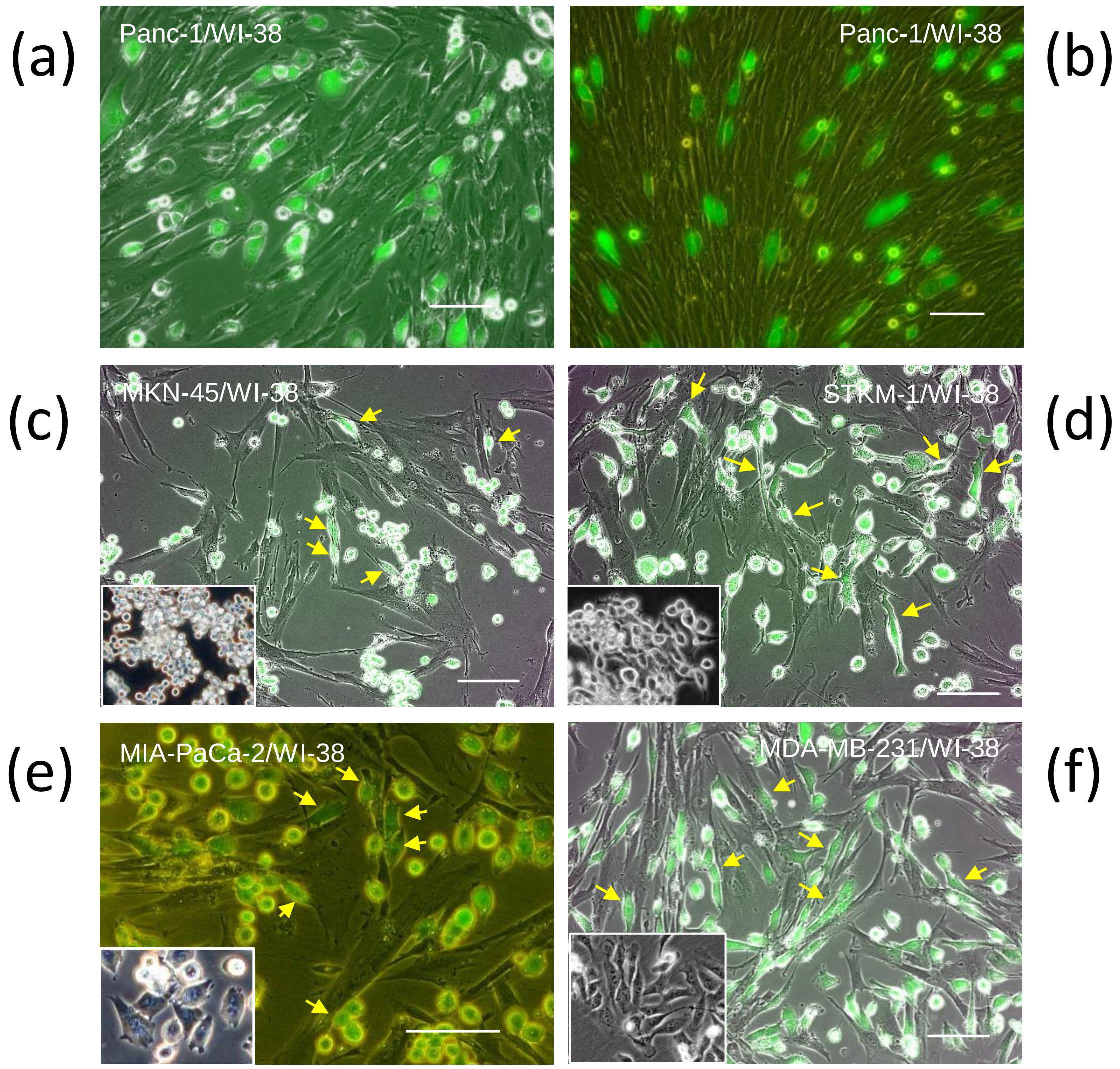

(a)
Panc-1/WI-38
(b)
Panc-1/WI-38
(c)
MKN-45/WI-38
(d)
STKM-1/WI-38
(e)
MIA-PaCa-2/WI-38
(f)
MDA-MB-231/WI-38

# Fig. S3

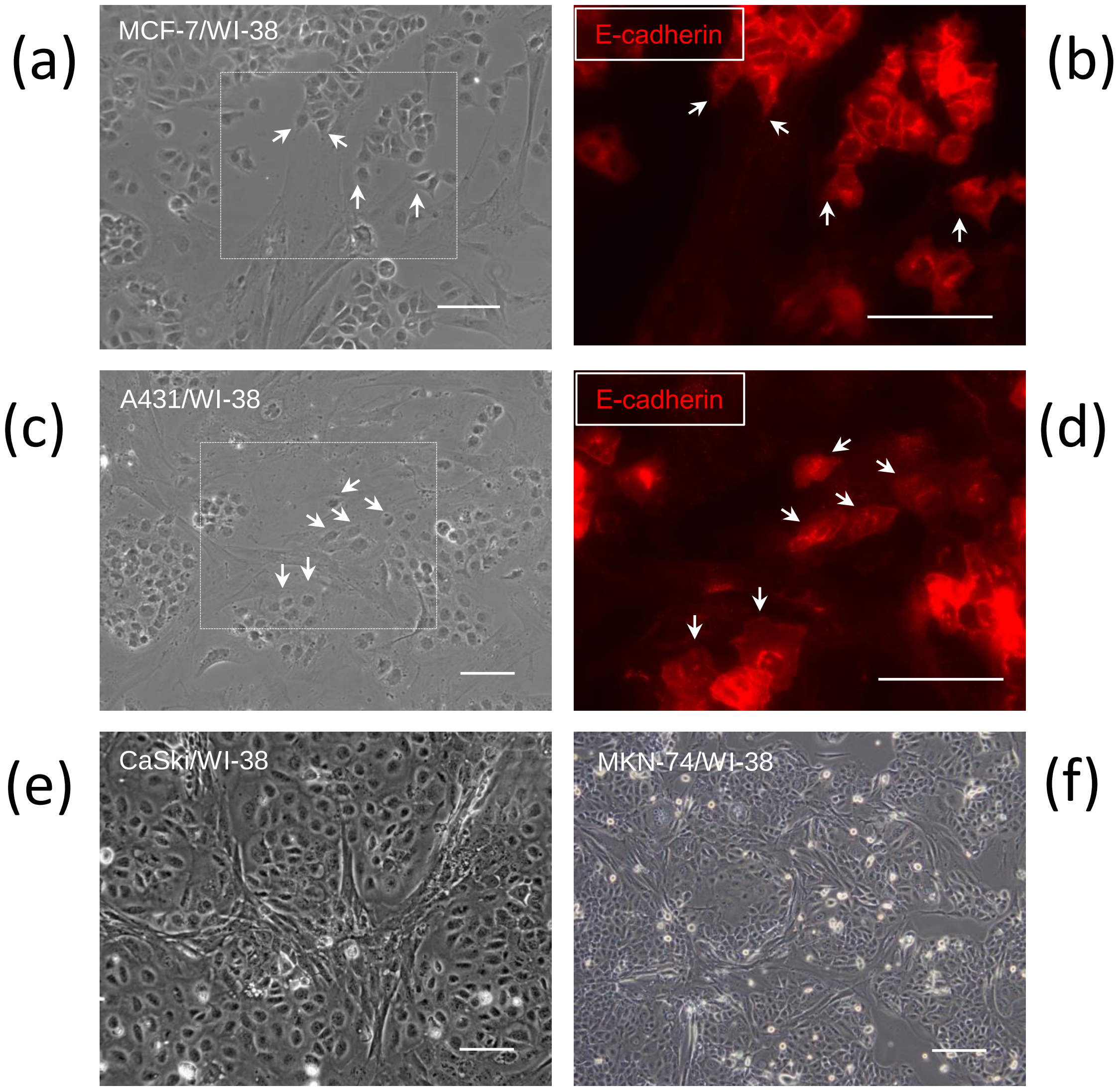
(a)
MCF-7/WI-38
(b)
E-cadherin
(c)
A431/WI-38
(d)
E-cadherin
(e)
CaSki/WI-38
(f)
MKN-74/WI-38

# Fig. S4

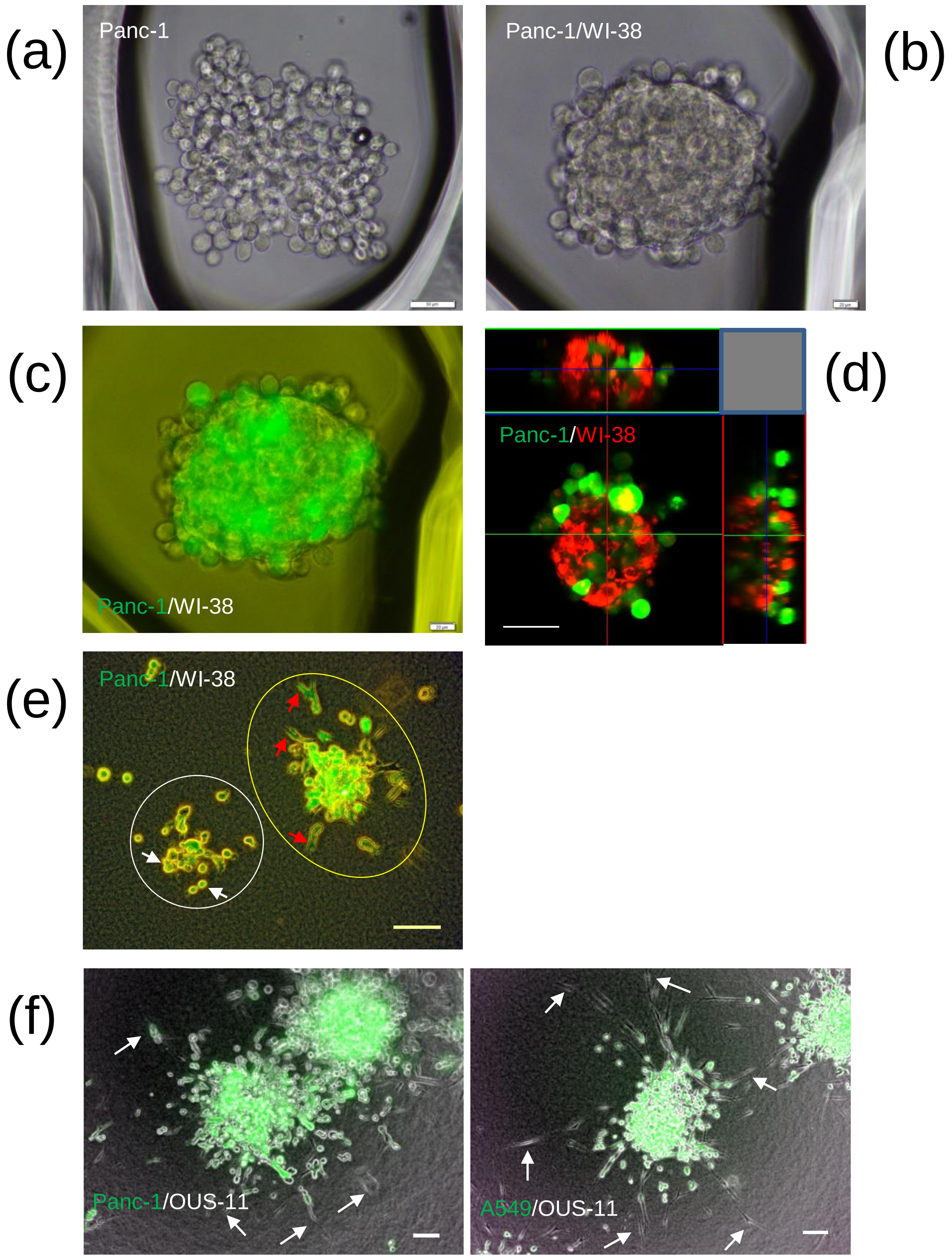

(a)
Panc-1
(b)
Panc-1/WI-38
(c)
Panc-1/WI-38
(d)
Panc-1/WI-38
(e)
Panc-1/WI-38
(f)
Panc-1/OUS-11
A549/OUS-11

# Fig. S5

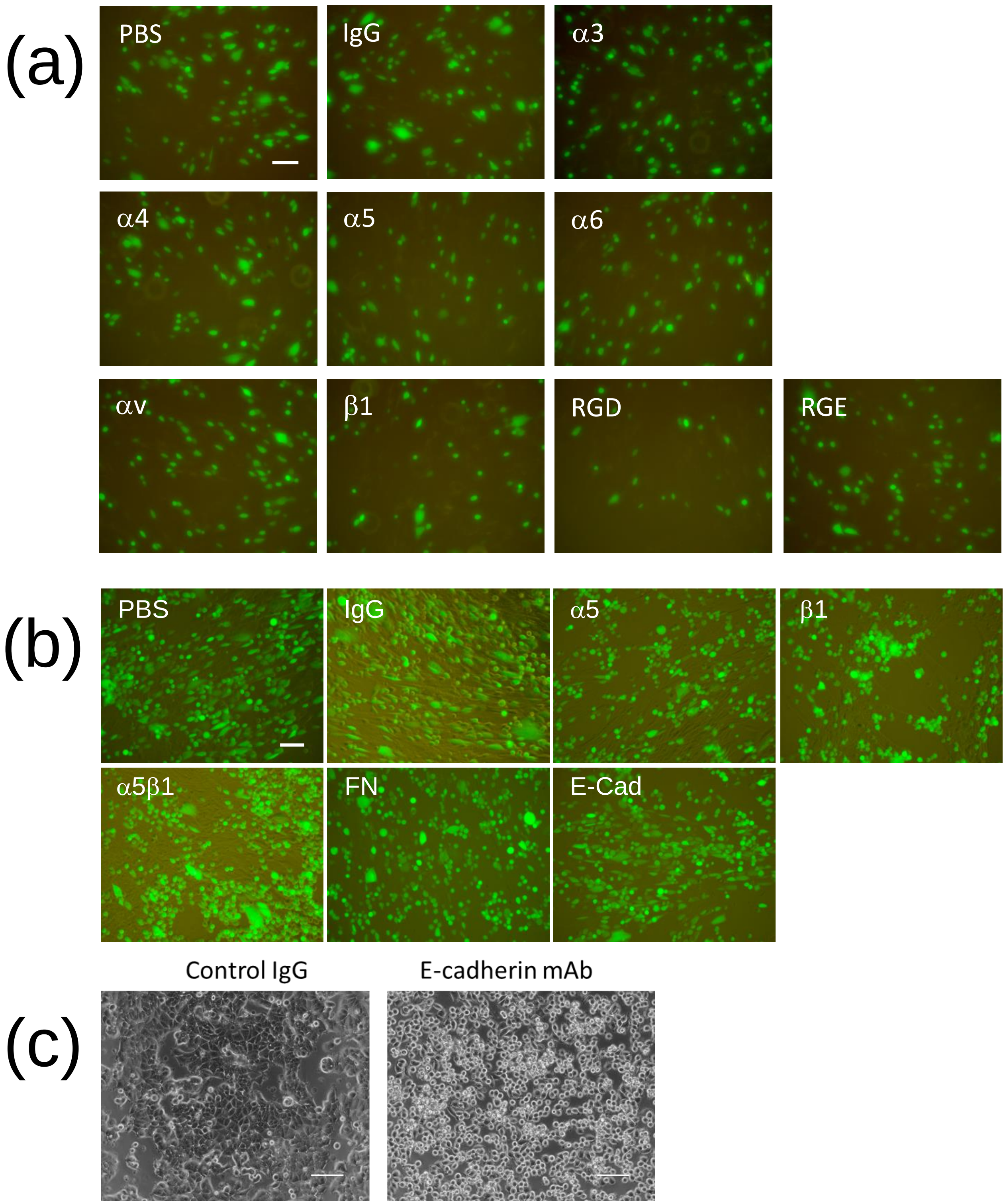
(a)
PBS
IgG
α3
α4
α5
α6
αv
β1
RGD
RGE
(b)
PBS
IgG
α5
β1
α5β1
FN
E-Cad
(c)
Control IgG
E-cadherin mAb

# Fig. S6

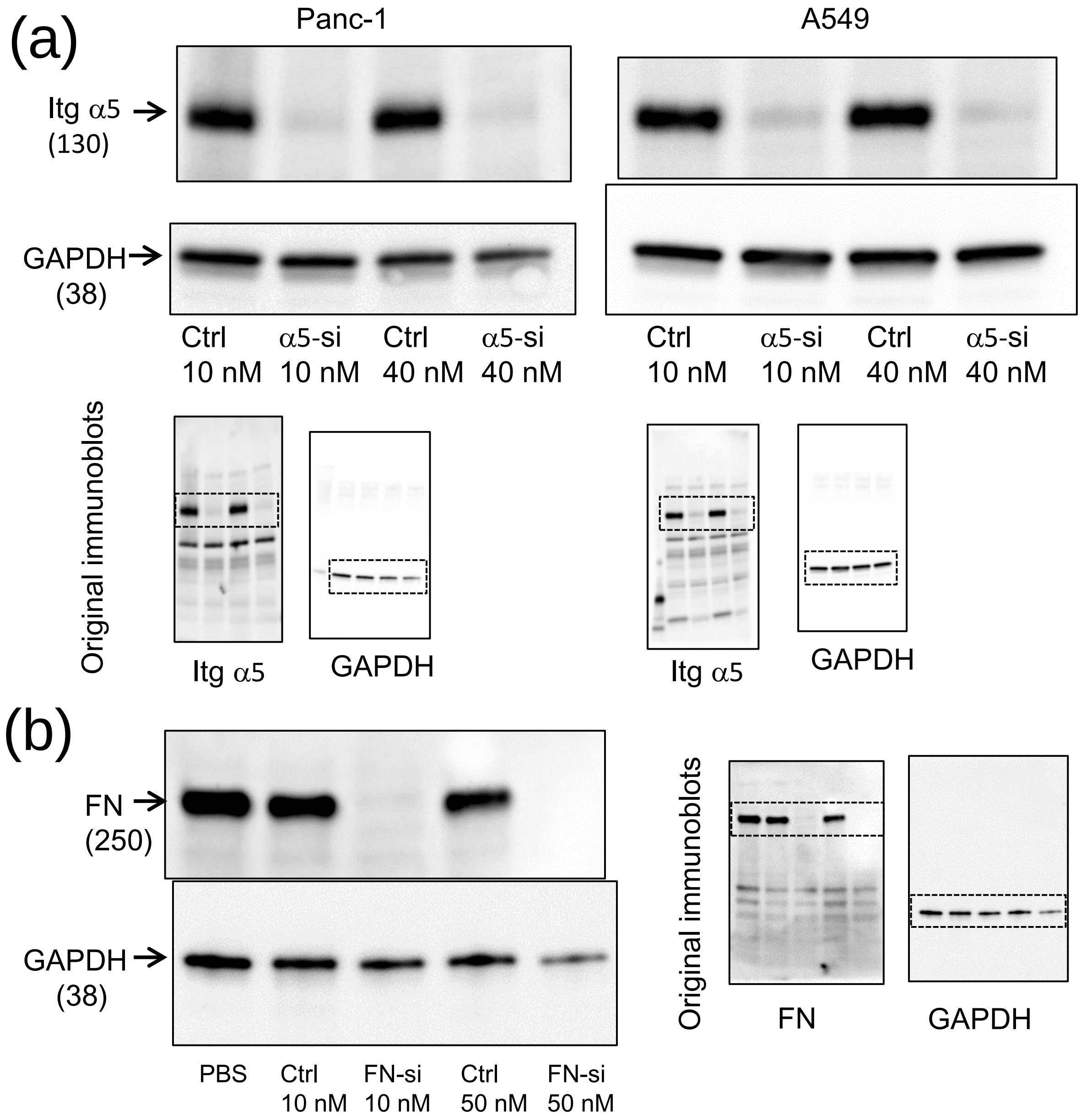
(a)
Panc-1
A549
Itg α5
(130)
GAPDH
(38)
Ctrl 10 nM
α5-si 10 nM
Ctrl 40 nM
α5-si 40 nM
Ctrl 10 nM
α5-si 10 nM
Ctrl 40 nM
α5-si 40 nM
Original immunoblots
Itg α5
GAPDH
Itg α5
GAPDH
(b)
FN
(250)
GAPDH
(38)
PBS
Ctrl 10 nM
FN-si 10 nM
Ctrl 50 nM
FN-si 50 nM
Original immunoblots
FN
GAPDH

# Fig. S7

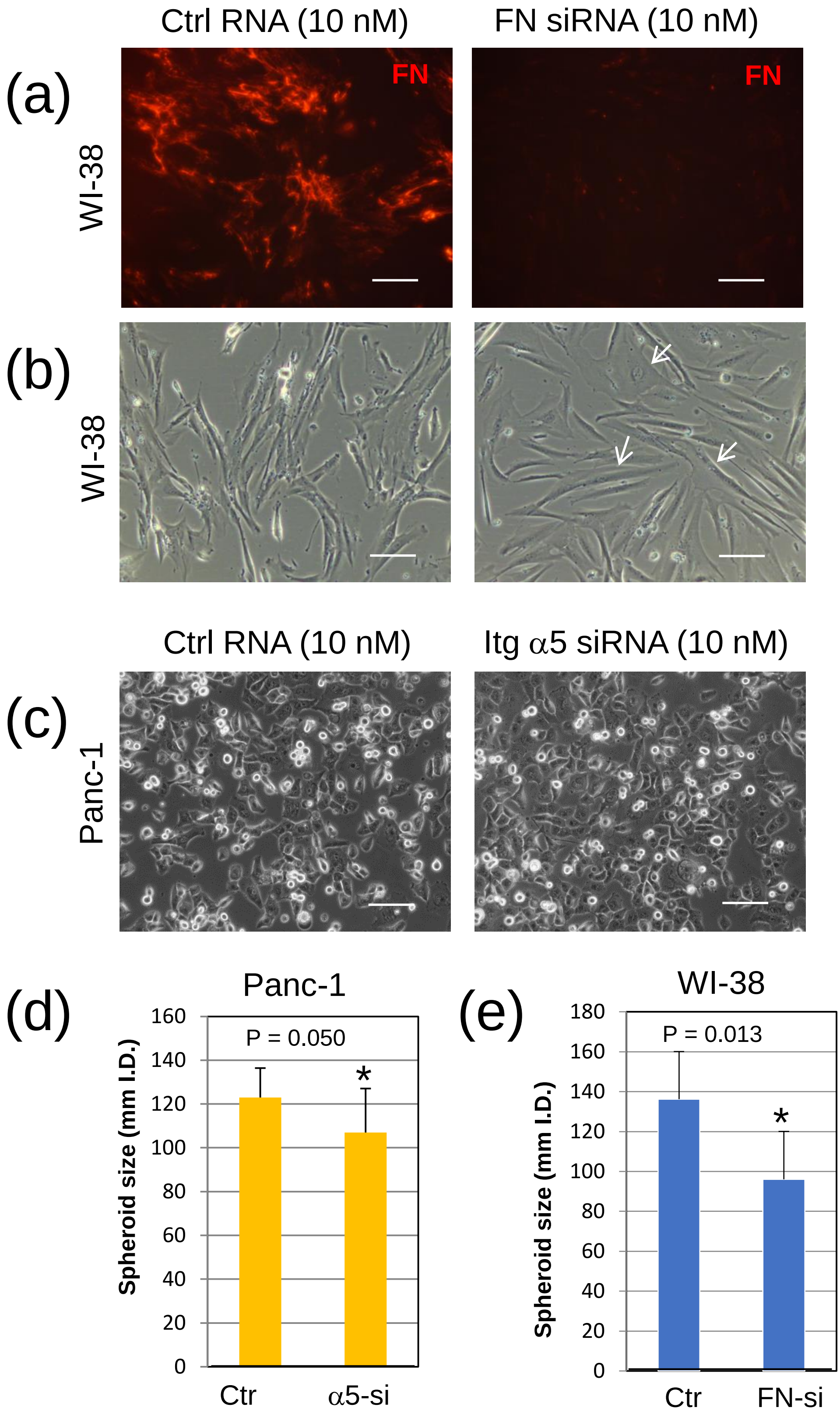
Ctrl RNA (10 nM)
FN siRNA (10 nM)
(a)
WI-38
FN
FN
(b)
WI-38
Ctrl RNA (10 nM)
Itg α5 siRNA (10 nM)
(c)
Panc-1
(d)
Panc-1
Spheroid size (mm I.D.)
P = 0.050
*
0
20
40
60
80
100
120
140
160
Ctr
α5-si
(e)
WI-38
Spheroid size (mm I.D.)
P = 0.013
*
0
20
40
60
80
100
120
140
160
180
Ctr
FN-si

# Fig. S8

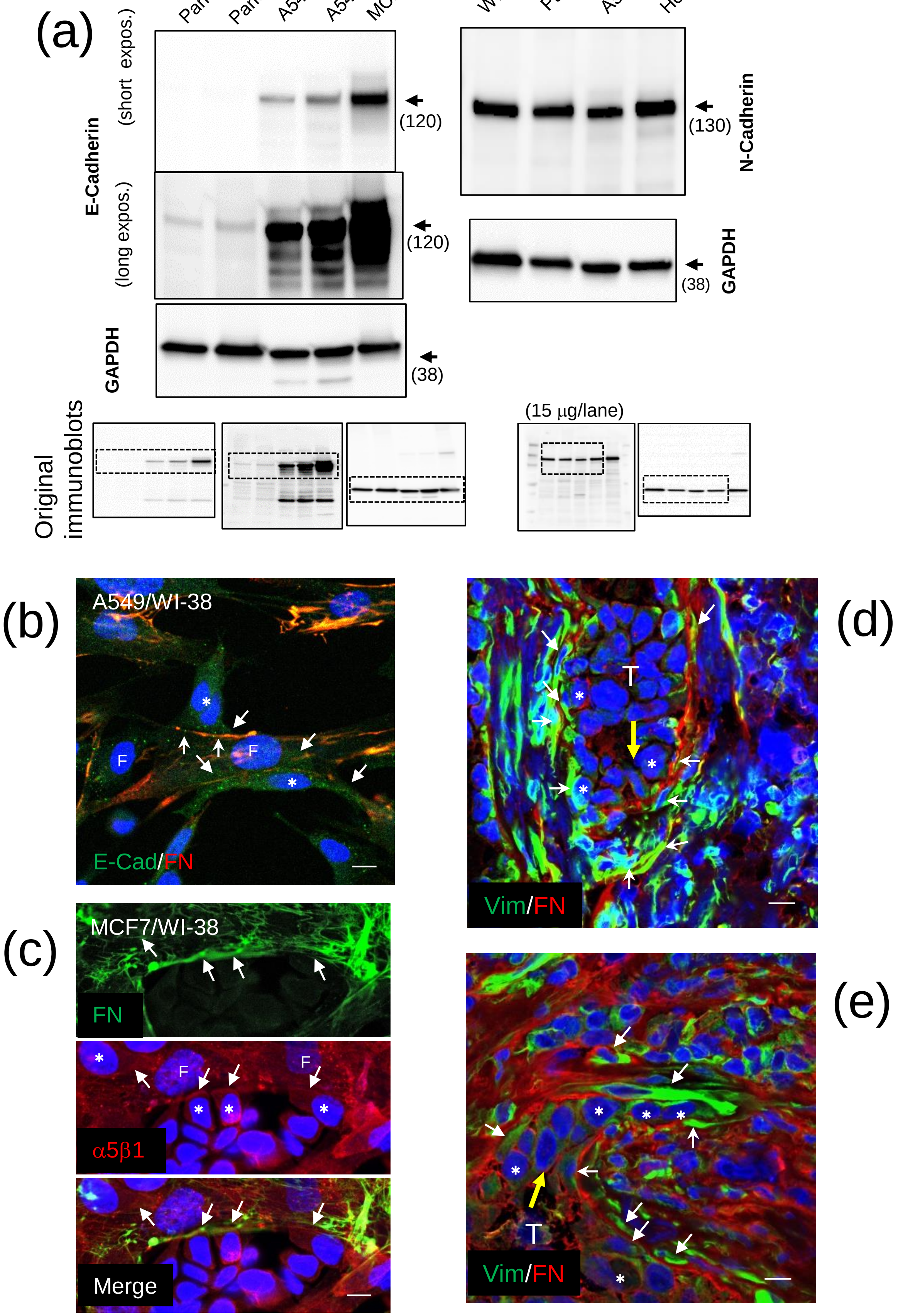

(a)
Panc-1 (15µg)
Panc-1 (30µg)
A549 (15µg)
A549 (30µg)
MCF7 (15µg)
E-Cadherin
(short expos.)
(long expos.)
(120)
(120)
GAPDH
(38)
WI-38
Panc-1
A549
HeLa
(130)
N-Cadherin
(38)
GAPDH
Original immunoblots
(15 µg/lane)
(b)
A549/WI-38
F
F
E-Cad/FN
(c)
MCF7/WI-38
FN
F
F
α5β1
Merge
(d)
T
Vim/FN
(e)
T
Vim/FN
